# N, N-Dimethyltryptamine, a natural hallucinogen, ameliorates Alzheimer’s disease by restoring neuronal Sigma-1 receptor-mediated endoplasmic reticulum-mitochondria crosstalk

**DOI:** 10.1186/s13195-024-01462-3

**Published:** 2024-05-01

**Authors:** Dan Cheng, Zhuo-Gui Lei, Kin Chu, Oi Jin Honey Lam, Chun Yuan Chiang, Zhang-Jin Zhang

**Affiliations:** 1 Department of Chinese Medicine, The University of Hong Kong-Shenzhen Hospital (HKU-SZH), Shenzhen, China; 2 School of Chinese Medicine, LKS Faculty of Medicine, The University of Hong Kong, Hong Kong, China; 3 Department of Neuroscience, City University of Hong Kong, Hong Kong, China; 4 Department of Psychology, LKS Faculty of Medicine, The University of Hong Kong, Hong Kong, China; 5 School of Biomedical Sciences, Faculty of Science, The University of Hong Kong, Hong Kong, China; 6 Digital Centre of State Key Laboratory of Quality Research in Chinese Medicine, Macau, China

**Keywords:** N,N-Dimethyltryptamine, cognitive impairment, Alzheimer’s disease, ER-mitochondria crosstalk, Sigma-1 receptor, calcium homeostasis

## Abstract

**Background:**

Aberrant neuronal Sigma-1 receptor (Sig-1r)-mediated endoplasmic reticulum (ER)- mitochondria signaling plays a key role in the neuronal cytopathology of Alzheimer’s disease (AD). The natural psychedelic N, N-dimethyltryptamine (DMT) is a Sig-1r agonist that may have the anti-AD potential through protecting neuronal ER-mitochondrial interplay.

**Methods:**

3×TG-AD transgenic mice were administered with chronic DMT (2 mg/kg) for 3 weeks and then performed water maze test. The Aβ accumulation in the mice brain were determined. The Sig-1r level upon DMT treatment was tested. The effect of DMT on the ER-mitochondrial contacts site and multiple mitochondria-associated membrane (MAM)-associated proteins were examined. The effect of DMT on calcium transport between ER and mitochondria and the mitochondrial function were also evaluated.

**Results:**

chronic DMT (2 mg/kg) markedly alleviated cognitive impairment of 3×TG-AD mice. In parallel, it largely diminished Aβ accumulation in the hippocampus and prefrontal cortex. DMT restored the decreased Sig-1r levels of 3×TG-AD transgenic mice. The hallucinogen reinstated the expression of multiple MAM-associated proteins in the brain of 3×TG-AD mice. DMT also prevented physical contact and calcium dynamic between the two organelles in in vitro and in vivo pathological circumstances. DMT modulated oxidative phosphorylation (OXPHOS) and ATP synthase in the in vitro model of AD.

**Conclusion:**

The anti-AD effects of DMT are associated with its protection of neuronal ER-mitochondria crosstalk via the activation of Sig-1r. DMT has the potential to serve as a novel preventive and therapeutic agent against AD.

**Supplementary Information:**

The online version contains supplementary material available at 10.1186/s13195-024-01462-3.

## Introduction

The escalating incidence of Alzheimer’s disease (AD) has posed a great challenge to the individuals, families, and societies [1]. Although researchers have made numerous attempts in the development of pharmacological drugs for AD, the clinical outcomes have been unsatisfactory. This is largely due to the fact that most approved drugs merely targeted the downstream pathways that restore acetylcholine (ACh) transmission and eliminate pathological products, mainly amyloid β (Aβ) and tau [2]. There have been various subcellular pathological alterations occurred in neuronal organelles that are closely associated with the upstream pathways of Aβ and tau accumulations [3]. The most apparent is aberrant signalling between the endoplasmic reticulum (ER) and mitochondria [3]. ER-mitochondria interplay plays an essential role in various neuronal functions, e.g., lipid metabolism, calcium homeostasis, autophagy, ER stress responses, axonal transport, and synaptic plasticity [4]. It is heavily involved in the pathogenesis of AD [4, 5]. This has led to the assumption that agents capable of restoring neuronal ER-mitochondria crosstalk may have anti-AD potentials.

ER-mitochondrial interface involves in specialized subdomains of the ER, known as mitochondria-associated membranes (MAMs) that are reversibly tethered to mitochondria [6]. The signalling between the two organelles is primarily achieved by releasing calcium from inositol 1,4,5-trisphosphate receptors (IP3Rs) of the ER to mitochondria, where calcium is taken into via voltage dependent anion channel 1 (VDAC1) and mitochondrial calcium uniporter (MCU), which are located in the outer mitochondrial membrane and inner mitochondrial membrane, respectively [4]. On the other hand, an ER protein, Sigma-1 receptor (Sig-1r), acts as a chaperone for IP3Rs to regulate calcium release from the ER to mitochondria, bioenergetics, ER stress, and cell differentiation [7–10]. Sig-1r is widely expressed throughout the central nervous system, with a high density in brain regions associated with learning and memory, such as the hippocampus and cortex [11–13]. Sig-1r activation inhibited Aβ deposition [14], whereas Sig-1r deactivation resulted in the development of AD [14, 15]. Several Sig-1r agonists are suggested to have benefits in alleviating neurodegenerative diseases in cultured cells and animal models [16]. These findings indicate that Sig-1r agonists may have the anti-AD potentials.

N, N-dimethyltryptamine (DMT) is a natural hallucinogenic alkaloid richly existing in Ayahuasca, an entheogenic beverage traditionally used in South Americans [17, 18]. While DMT acts at 5-HT_2A_ receptors as agonist, it is also a Sig-1r agonist and has been proven to have potent protective effects against hypoxia and cerebral ischemia by activating Sig-1r [17, 19, 20]. DMT enhanced spatial learning and memory tasks by promoting adult neurogenesis via Sig-1r, but not 5-HT_1A_ and 5-HT_2A_ receptors [21]. Intravenous injection of DMT produced antidepressant efficacy in people with treatment-resistant depression [22]. These studies imply that the psychotropic effects of DMT may be related to the activation of Sig-1r, which restores ER-mitochondria signalling. Unlike most hallucinogenic drugs, DMT did not cause or caused less tolerance, dependence, and withdrawal symptoms [23]. It thus deserves to be further developed as a novel anti-AD agent.

The working hypothesis of this study was that DMT exerted anti-AD effects by restoring neuronal ER-mitochondria signaling via the Sig-1r activation. To test this hypothesis, we firstly examined the effects of DMT chronic treatment on cognitive impairment and Aβ deposits in transgenic 3×TG-AD mice, one of animal models of AD. We then evaluated the effects of DMT on the level of Sig-1r and MAM-associated proteins, physical contact, calcium dynamic between ER and mitochondria in in vitro and in vivo paradigms, and oxidative phosphorylation (OXPHOS) and ATP synthase with an additional utilization of Sig-1r knockdown mice and the Sig-1r antagonist BD1063.

## Methods

### Animals

Forty-eight 3×TG-AD transgenic mice were used to mimic Alzheimer’s disease in this study. The 3×TG-AD strain [B6; 129-Tg (APPSwe, tau P301L) 1LfA Psen1 tm1Mpm /Mmjax] is a unique animal model that features three human mutant genes (APPswe, PSEN1, and tauP301L) and exhibits Aβ and tau pathology [24]. Twenty-four congenic C57BL/6J wild-type mice served control. All mice used in experiments aged 8-10 months and weighed 25-35 g. The research protocols and animal care were approved by the Committee on the Use of Live Animals in Teaching and Research of the University of Hong Kong (CULATR No.: 5345-20) and performed following the guidelines.

### Preparation and administration of drugs

DMT was obtained from Sigma-Aldrich, USA. For animal study, DMT was dissolved ultrasonically in Phosphate-buffered saline (PBS) to a stock concentration of 1.2 mg/ml and then freshly diluted to a final dosage of 2 mg/kg. The transgenic and wild-type (WT) mice received 2 mg/kg of DMT or equivalent volume of saline (vehicle) via intraperitoneal injection per day for 3 weeks. For in vitro experiments, DMT, Aβ_25–35_ oligomers, and the Sig-1r antagonist BD1063 were used. The Aβ_25-35_ oligomers was generated as we previously described [25]. Briefly, Aβ_25–35_ monomer was dissolved in the sterile distilled water to a stock concentration of 1 mmol/L and then incubated at 37^°^C for 7 days. Primary hippocampal neurons were exposed to 1 μM BD1063 and 20 μM Aβ for 24 h with or without DMT(1 μM) treatment (Fig. S1, S2).

### Establishment of Sig-1r knockdown mice

In order to determine the role of Sig-1r in the effects of DMT on neuronal ER-mitochondria crosstalk, Sig-1r knockdown mice were developed using the short-hairpin RNA (ShRNA) technique. Custom-made adeno-associated virus (AAV) vectors carrying ShRNA (sh-Sig1r) and a AAV control (nc-Sig1r) were obtained from OBiO technology (Shanghai, China). Following one week of habituation, mice were anesthetized with ketamine/xylazine, eye dehydration was prevented by the topical application of the ophthalmic gel, and then placed on a stereotaxic apparatus with a mouse adaptor and ear bars (RWD Instruments, China). A 3-mm craniotomy was performed bilaterally with a pneumatic dental drill, leaving the dura intact. The bregma and lambda are then aligned to target the hippocampus under the following coordinates: anterior-posterior, -1.85; medial-lateral, ±1.10 mm; and dorsal-ventral, -2.04. The nc-Sig1r and sh-Sig1r were bilaterally injected into the hippocampi using a Hamilton microliter syringe at a rate of 50 nl/min and a volume of 150 nl. Following the completion of the injection, the virus was allowed to diffuse for at least 10 min before the pipette was slowly withdrawn. Mice were allowed to recover for 3-4 weeks.

### Water maze test for spatial memory

Water maze test is a valid behavioral paradigm for the study of spatial learning and memory [26]. There were 8 mice with equal number of male and female mice per group in the test. The test was performed at the end of 3 weeks treatment in mice as our previous description [27]. Briefly, during the 5-day training phase, the platform was placed in the centre of the target quadrant and submerged under 1 cm of the water surface. The escape latency of mice to find the hidden platform was recorded after being released from the randomly selected quadrant. Mice that failed to located on the platform within 60 s were placed on the platform and remain for 30 s. The training trial was conducted twice daily, and the average of the two trials was presented. During a 60 s probe test on the 6 th day, the platform was removed and the mice were allowed to find the target quadrant and the original platform. The swimming path of the individual mouse was recorded using the SMART video tracking system (V3, Panlab, Spain).

### Thioflavin S staining for Aβ deposits

Thioflavin S, a fluorescent dye, was widely used as a standard staining method for Aβ plaques. Brain slices (n = 3 each group from behavioral tests) were stained with Thioflavin S as previously described [28]. Briefly, brain slice sections were dehydrated in a series of gradient ethyl alcohol solutions (100%, 95%, 80%, 50%) for 3 min per solution and rehydrated in distilled water 3 times for a few seconds in each time. The sections were then stained for 10 min with 1% Thioflavin S and dehydrated with a gradient ethyl alcohol solutions (50%, 80%, 95%, 100%) with 3 min in each one. Finally, the slides were submerged in xylene for 3 min before being covered with mounting media. The stained section was observed on a confocal laser-scanning microscope (Zeiss microscope 880). The results were presented as the mean area of the Thioflavin S-positive Aβ deposits in the total field area.

### Transmission electron microscopic detection of ER-mitochondrial distance

Following the vehicle and DMT treatment, animals (n = 3 each group, some from behavioral tests) were perfused with PBS, the hippocampi were dissected and then fixed in a 2.5% glutaraldehyde solution overnight at 4°C. The hippocampal tissues were washed in PBS, subsequently post-fixed and stained. The sections were imaged on an electron microscope (Philips). Morphometry was done to measure ER-mitochondria distance using FIJI software [29]. At least 5 mitochondria were randomly chosen from each image for 10 images each animal. The distances were computed within a field of an approximately 30-nm radius from a chosen mitochondrion.

### Analysis of MAM-associated proteins

Multiple MAM-associated proteins, in particular p*hosphofurin acidic cluster sorting protein 2 (*PACS2), phosphatidylserine synthase 1 (PSS1), mitofusin 2 (MFN2), sigma-1 receptor (Sig-1r), vesicle-associated membrane protein-associated protein B (VAPB), and C/EBP Homologous Protein (CHOP), play a crucial role in the maintenance of calcium homeostasis [30–32]. The effects of DMT on these proteins were examined using Western blot in the whole brain (cerebellum excluded) (n = 3 each group). Our preliminary experiments revealed that the expression of most of the 6 aforesaid proteins was low and even undetectable in the hippocampi.

#### MAM purification

MAM was purified as previously described with optimization [33]. Briefly, mice brains were homogenized using a LabServ Homogenizer (Thermo Fisher Scientific, USA). The homogenate was then centrifuged at 800 × g in order to remove the debris and nuclei. Next the supernatant was centrifuged at 9,000 × g, the crude mitochondrial pellets were collected, and subsequently were suspended in 2 ml of a re-suspension buffer containing 250 mM mannitol, 5 mM HEPES, and 0.5 mM EGTA. Then layered the re-suspension buffer on top of 30% percoll medium (225 mM mannitol, 25 mM HEPES, and 1 mM EGTA) and centrifuged at 100,000 × g for 30 min to separate mitochondria and MAM. The fraction of MAMs was extracted and collected from the upper band, subsequently centrifuged at 9,000 × g and 100,000 × g to remove the contaminants and mitochondrial contamination in the MAM fraction. The purified MAM was preserved at -80°C for later use.

#### Western blot analysis

The purified MAM was lysed in RIPA buffer plus the protease inhibitor. 30 μg of total proteins from the MAM were separated by 10% sodium dodecyl sulfate-polyacrylamide gel electrophoresis (SDS-PAGE) and transferred to polyvinylidene difluoride (PVDF) membranes following protein determination. The membranes were then incubated with 5% BSA for 1.5 hours, followed by the primary antibodies against PACS2, PSS1, MFN2, Sig-1r, VAPB, and CHOP. The detailed information of the antibodies was shown in Table S1. The secondary antibodies were then incubated for 1.5 hours. The quantitative expression level of proteins was determined using the ChemiDoc XRS+ imaging system (Bio-Rad, USA) after being detected with an ECL select detection reagent (GE Healthcare, USA).

### Immunofluorescent detection of effects of DMT on Sig-1r-lablled neurons

The effect of DMT on Sig-1r-lablled neurons in the in vivo studies was determined by using immunofluorescent staining. Mice were euthanized and perfused with 4% paraformaldehyde (PFA) following PBS. The whole brains were removed and kept overnight in 4% PFA at 4°C. Then the brains were immersed in of 30% sucrose for 2-3 days. 30 μm thick coronal slices were cut using a cryomicrotome (Leica, Germany) for immunofluorescent staining. The brain sections were blocked for 1 hour with a blocking buffer containing 5% BSA/0.3% Triton X-100. Then the sections were incubated with primary antibodies against Sig-1r (1:200, Abcam) overnight at 4°C. After washing, the sections and cells were incubated for 1.5 h with the fluorescence-conjugated secondary antibodies and then stained with 4′,6-diamidino-2-phenylindole (DAPI). The sections and cells were visualized using a confocal microscope (Zeiss LSM880). The fluorescence intensity of Sig-1r were count on an average of 5 randomly selected sections from each mouse (n = 3 per group). The ratio to the control group was calculated.

### Quantitative polymerase chain reaction

Quantitative polymerase chain reaction (qPCR) was used to  measure the mRNA level of the Sig-1r in the mice hippocampus as we previously described [27]. Briefly, 1 μg of total RNA was reversed transcribed into first-strand cDNA using a PrimeScript RT Master Mix (TaKaRa, Japan), and then the PCR amplification was performed with SYBR Green PCR Kit Mix ((QIAGEN, USA) with following parameters: 95°C (15 min), followed by 40 cycles of 95°C (15s), 60°C (30 s). Relative quantification of the mRNA in the group of Sig-1r knockdown mice was calculated using 2^−∆∆ct^ and expressed as a percentage of negative control group. The primer sequences were shown as following: β-actin-F: 5’-CCACCCTGAAATCCTGTC CA-3’; β-actin-R: 5’-TGAAGCAAA GGCCAGGCTAA-3’; Sig-1r-F: CATTTGGTGTCTAAGCGCGA; Sig-1r-R: AAGCCAGCCTCAGATTTCTC.

### Primary hippocampal neuron culture

Hippocampi were dissected from C57BL/6J pups at P0-1 under a dissection microscope. Neuronal cells were washed 3 times in Hank’s balanced salt solution (HBSS). The enzymatic digestion procedure was performed following the manufacture’s protocol of the Neural Tissue Dissociation Kit (P) (Miltenyi Biotec, USA). After centrifugation at 300 RCF for 10 min at 4°C, the supernatant was removed. Cells were re-suspended in a neurobasal medium containing additional of 2% B27, 1% GlutaMAX, 20 μg/ml β-fibroblast growth factor (β-FGF), 20 μg/ml epidermal growth factor (EGF) and 1% penicillin/streptomycin (50 U/ml). Then the resuspended cells were seeded on the Poly-D-lysine (PDL)-coated plates and cultured in a humidified 95% air and 5% CO_2_ atmosphere at 37°C for 7 days. On day 3, a final dose of 10 μM Ara-C (Sigma, USA) was administered to prevent glia overgrowth [34].

### Proximity ligation assay (PLA) of ER-mitochondrial contacts in cultured neurons

IP3R3 and VDAC1 are markers of the ER-mitochondrial contacts and detected using the Duolink Proximity Ligation Assay (Sigma-Aldrich, USA) according to the manufacturer’s instruction. Briefly, primary hippocampal cells were seeded on the 24-well plates with a 10 mm coverslip and incubated for 7 days, then cells were exposed to Aβ_25-35_ or BD1063 at the final concentrations of 20 μM and 1 μM, respectively, with or without DMT treatment for 24 h. Following treatment, cells were fixed in 4% PFA for 10 min. After that, cells were incubated with 100 mM glycine for 15 min, subsequently incubated with 0.1% Triton X-100 in PBS for 15 min for permeabilization and blocked for 30 min with blocking buffer from the Duolink In Situ PLA Probe kit (Sigma-Aldrich, USA). The antibodies against IP3R3 (1:200, ABclonal) and VDAC1 (1:200, Sant Cruz Biotechnology) were co-incubated overnight at 4°C. Cells were washed twice with Duolink Fluorescent wash buffer A, then incubated with the Duolink In Situ PLA Probes for 1 h. After that, the ligation and amplification procedures were performed. Finally, cells were dried and mounted with DAPI-containing aqueous mounting media (Sigma-Aldrich, USA), and visualized using a confocal microscope (Zeiss LSM880) with a 40×oil immersion lens.

### Mitochondrial and cytosolic calcium imaging in cultured neurons

The mitochondrial matrix-targeted Ca^2+^ sensitive indicator, Mito-GCaMP6s (AAV2/9-hSyn-DIO-GCaMP6s.Mito-WPRE-pA) (Taitool Bioscience, Shanghai, China) was used to analyze mitochondrial matrix calcium. The cultured primary hippocampal neurons were seeded on 25-mm glass coverslips and incubated for 7 days before transfection. The transfection was performed following the manufacturer’s instructions of OPTI-MEM and Lipofectamine 2000 (Thermofisher Scientific, USA). Following transfection, the cells were grown for 48 h for optimal expression.

For cytosolic Ca^2+^ imaging, cells were stained with the cytosolic Ca^2+^ indicator, 5 μM Fluo-4AM (ThermoFisher Scientific), for 30 min at 37 °C with the Hepes-buffered Hanks’ balanced salt solution (Hepes-HBSS, 137 mM NaCl, 5.4 mM KCl, 2 mM CaCl_2_, 0.25 mM Na_2_HPO_4_, 0.44 mM KH_2_PO_4_, 5.5 mM glucose, 4.2 mM NaHCO3, 10 mM HEPES, pH 7.4) [35]. After Fluo-4AM loading or expression of GCaMP6, cells were washed and incubated with the Hepes-HBSS, and then placed in an open bath imaging chamber for image acquisition. Time-lapse Ca^2+^ images were obtained every 1 s for 5 min using a Nikon Ti2-E microscope with a 20 × objective lens. 1 min after image acquisition, 100 μM ATP was added to cells to induce Ca^2+^ release from ER-stores [36].

Data analysis for the calcium signal was performed using MetaMorph software [37, 38]. The fluorescence signals were measured by averaging intensities of the circular regions of interest (ROI). Ca^2+^ fluctuations were quantified as △F/F0. F0 is the average baseline fluorescence intensity before ATP stimulation, and △F is the baseline subtracted fluorescence intensity.

### Measurement of mitochondrial respiration

Mitochondrial respiration was measured using Seahorse XF Cell Mito Stress Test Kit (Agilent Technologies, USA) in a Seahorse XFe96 Analyzer (Seahorse Bioscience, USA) according to the manufacturer’s instructions. The effects of DMT on several mitochondrial respiration parameters (basal and maximal respiration, proton leak, and ATP-linked respiration) were obtained. The effects of DMT under various stressors [2 μM oligomycin, 0.5 μM carbonilcyanide *p*-triflouromethoxyphenylhydrazone (FCCP), and 1 μM rotenone/antimycin A] were tested. Cultured cells were seeded at a density of 8×10^3^ cells/well, grew for 7 days, and exposed to 20 μM Aβ_25-35_ in the presence or absence of 1 μM DMT for 24 h. Upon measurement, cultured cells were washed twice and maintained in an XF assay medium. The cells were incubated at 37°C for 30-60 min in a non-CO_2_ incubator. The aforesaid stressor solutions were sequentially added into the wells of a utility plate. Oxygen consumption rate (OCR) is respective indicators of OXPHOS of the tricarboxylic acid (TCA) cycle. The OCR rate was calculated and expressed as pmol/min. Data were analyzed using Seahorse XF96 Wave software.

### Measurement of the mitochondrial membrane potential

Mitochondrial membrane potential (MMP) is an important component of intermediates during OXPHOS required for ATP synthesis [39]. MMP was analysed by flow cytometry with a JC-1 mitochondrial assay kit (Beyotime Biotechnology, China) in this study. Cells were washed twice in PBS and incubated at 37°C for 30 min at the final concentration of JC-1 (1×) dissolved in the staining buffer and distilled water. The cells were washed in PBS and re-suspended in the same buffer in the culture tube for flow cytometry detection.

### Measurement of intracellular ATP level

The intracellular ATP level in primary hippocampal cells was measured using a luminescent ATP detection assay kit (Abcam, USA). Cultured cells were plated into 6-well plates at a density of 1×10^5^ cells/ml and cultured for 7 days. Following treatment with Aβ_25-35_ and DMT as described above, the medium was discarded; the cells were washed three times in PBS and lysed for 30 min by adding 200 μl lysis buffer to each well. The cells were harvested and centrifuged at 12,000 g for 5 min at 4°C; the supernatant was aspirated for cell lysate. Finally, 90 μl ATP assay solution was added to 10 μl of cell lysate or the ATP standard solution in the 96-well plates. The emitted light was linearly related to the ATP concentration and measured with a fluorospectrometer.

### Statistical analysis

Statistical analysis and figure generation were performed using GraphPad Prism 9 (GraphPad Software, USA). Data was expressed as mean ± SE and analysed by One-way analysis of variance (ANOVA), two-way ANOVA analysis or T-test. The Tukey’s post hoc test was used to detect statistical significance between groups. All statistical tests were two-tailed. P-value < 0.05 was considered statistically significant.

## Results

### DMT treatment improved cognitive impairment and diminished Aβ pathology in 3×TG-AD mice

**Fig. 1:**
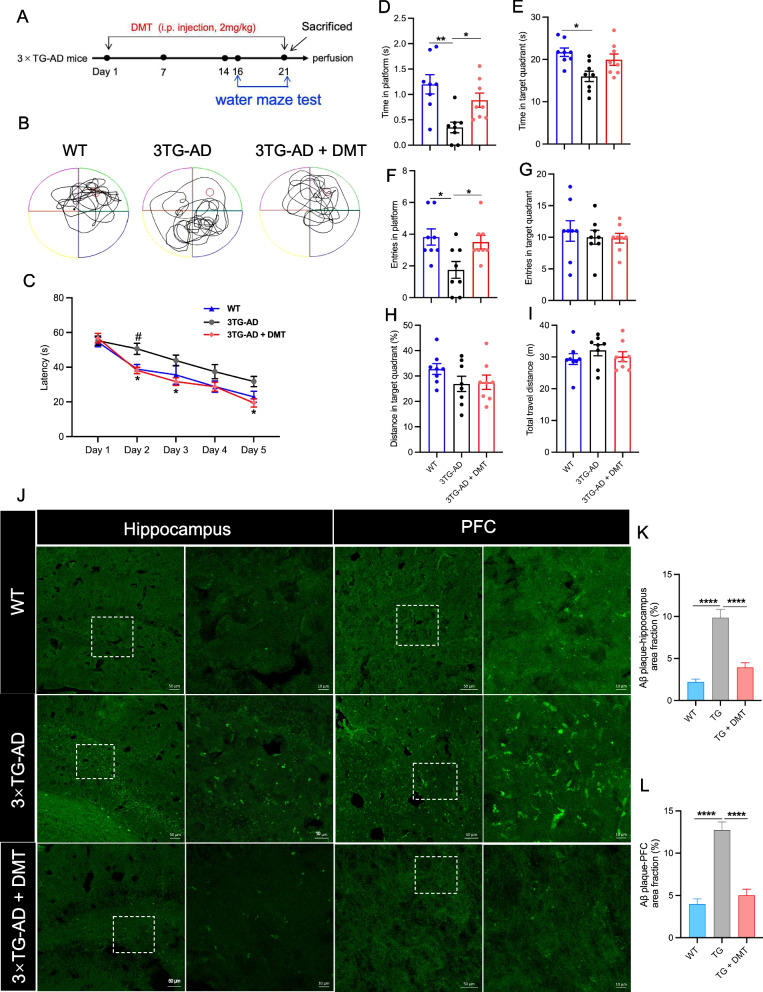
DMT improves cognitive impairment and diminishes Aβ pathology in the 3×TG-AD mice. **A** Experimental timeline. **B** The representative swim paths of mice. **C** Latency to platform of mice during the training trial. **D** Time in platform, **E** Time in target quadrant, F Entries in platform, **G** Entries in target quadrant, **H** Distance in target quadrant, **I** Total travel distance of mice in the probe test. (n = 8 in each group). **J** The representative images of Aβ plaque in the hippocampus and prefrontal cortex (PFC) of 3×TG-AD mice. The images on the right are enlarged views of the image on the left in each panel. Scale bar: 50 μm (left), 10 μm (right). **K**, **L** The quantification of the Aβ accumulation in the hippocampus and PFC, respectively. The results were presented as the mean area fraction of the positive particle. In the training trials of water maze test, data were analyzed using two-way ANOVA test; in the probe test, data were analyzed using one-way ANOVA. Data analysis of Aβ plaque was performed on average 5 randomly selected sections from each mouse (*n* = 3 per group) using one-way ANOVA. *P < 0.05, **P < 0.01, ****P < 0.0001. TG, 3×TG-AD

The spatial learning and memory of mice was evaluated in water maze test. In the training trials, the latency to find the platform in the 3×TG-AD mice injected with vehicle significantly prolonged on the 2nd day relative to the WT mice. While 3×TG-AD mice injected with DMT had a significant reduction in the latency at Day 2, 3, and 5 compared to the vehicle-treated animals (Fig. 1C). The performance of mice in the probe trail showed the behavioral variables, time spent in platform, target quadrant, and entries into platform largely decreased in the 3×TG-AD mice relative to WT mice (Fig. 1D-F). DMT treatment markedly increased time and entries in the platform in the model mice (Fig. 1D-F). No significantly statistical differences were observed in the DMT improvement on these two parameters between male and female mice (Fig. S4). The three groups did not differ in other parameters (time and entries in target quadrant, distance in target quadrant, and total travel distance) (Fig. 1G-I). These results suggested that chronic DMT treatment was sufficient to suppress cognitive decline of AD.

Next, we investigated whether Aβ plaques in the mice brain were diminished with DMT treatment by using Thioflavin-S staining (Fig. 1J-L). The transgenic mice had a strikingly higher density of Aβ plaques in the hippocampus and prefrontal cortex (PFC) regions than WT mice. DMT treatment significantly facilitated the degradation of Aβ plaques in these two regions.

### Effects of DMT on Sig-1r level in mice and cultured neurons

We found that compared with age-matched WT mice, the expression level of Sig-1r in the hippocampus of 3×TG-AD mice significantly decreased with the progression of AD (Fig. S3). This result was consistent with previous studies [5], and Sig-1r levels were also found to be reduced in the brains of AD patients [5, 40]. However, whether the reduced expression of Sig-1r was a result or one of the causes of AD remains for further investigation [41].

**Fig. 2:**
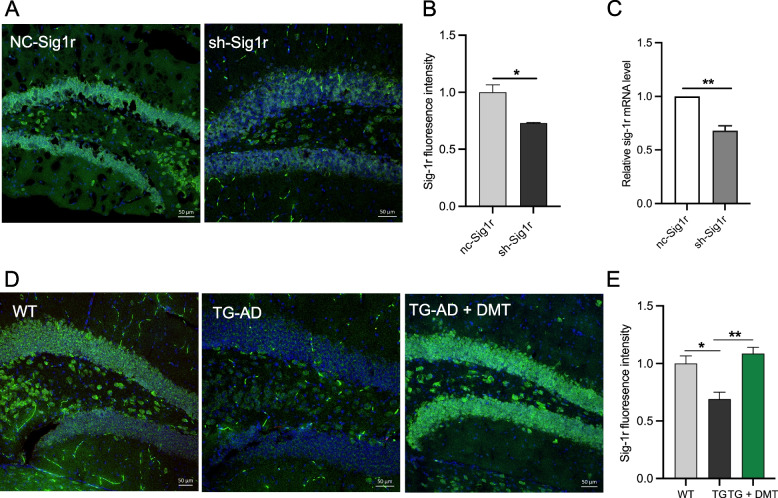
DMT improves the Sig-1r levels in the mice hippocampus and primary hippocampal neurons. **A**, **B** Representatives and the quantification of the Sig-1r level in the hippocampus of sh-Sig1r knockdown mice. **C** The mRNA level of Sig-1r in the hippocampus of sh-Sig1r knockdown mice. **D**, **E** Representative images and the quantification of the Sig-1r level in the hippocampus of 3×TG-AD mice. In the in vivo study, data was obtained from average 5 randomly selected sections from each mouse (*n* = 3 per group). Data was analyzed using one-way ANOVA. *P < 0.05, **P < 0.01. TG, 3×TG-AD

The effects of DMT on Sig-1r level was examined in the in vivo paradigms. The expression of Sig-1r level was determined in Sig-1r knockdown mice and 3×TG-AD mice using immunofluorescent staining. The Sig-1r knockdown mice exhibited a marked decrease in the Sig-1r expression in the mice hippocampus relative to the control (Fig. 2A-B**)**. The mRNA level of Sig-1r was further confirmed the decreased Sig-1r level in the Sig-1r knockdown mice (Fig.2C). The Sig-1r fluorescence intensity in the hippocampus of 3×TG-AD mice obviously decreased compared to WT mice. Chronic DMT administration completely reversed the expression of Sig-1r to the level in the control mice (Fig. 2D-E). Our findings indicated that low-dose chronic treatment with DMT increased Sig-1r levels in in vivo models of AD. These results were consistent with other previous studies, which found that the agonist of Sig-1r increased the expression of Sig-1r protein and mRNA [42, 43].

### Effects of DMT on neuronal ER-mitochondria distance in neuronal tissues

**Fig. 3:**
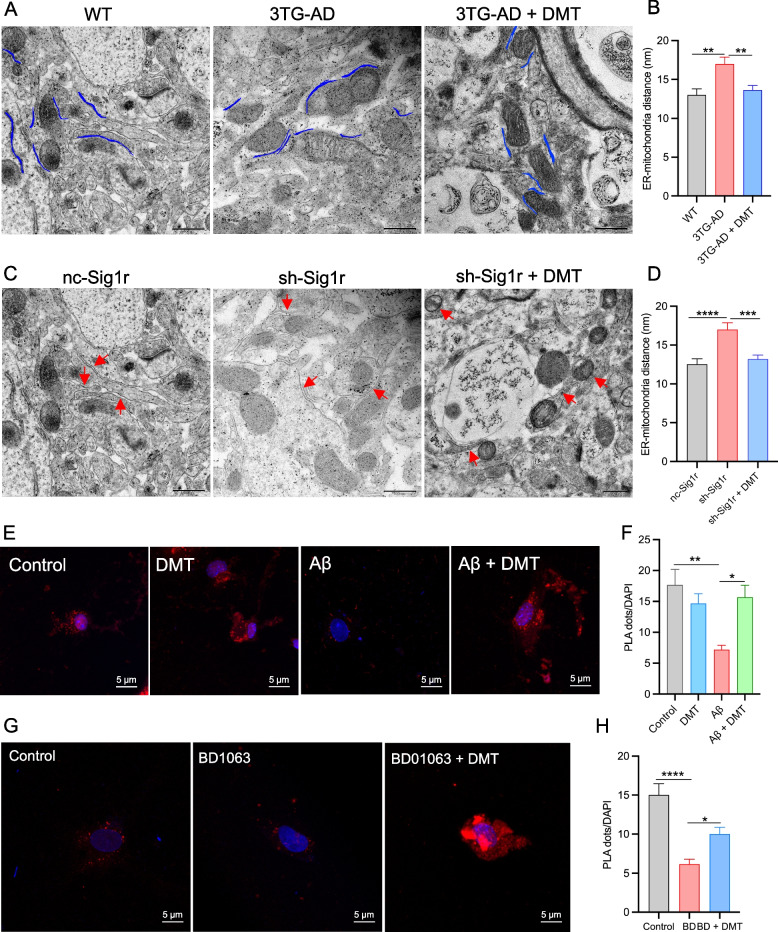
DMT modulates endoplasmic reticulum (ER) – mitochondrial distance and contacts. Two different animal models, 3×TG-AD mice and sh-Sig1r knockdown mice were used. **A**, **B** Representatives and quantification of ER-mitochondrial distance in the hippocampus of 3×TG-AD mice. **C**, **D** Representatives and quantitative analysis of ER-mitochondrial distance in the hippocampus of sh-Sig1r knock down mice. Scale bar: 500 nm. Data was obtained from 3 mice per group with 10 images in each mouse. **E**, **F** Representatives and quantification of the PLA signal in the cells exposed to Aβ with or without DMT treatment. **G**, **H** Representatives and quantification of the PLA signal in the cells exposed to BD1063 with or without DMT treatment. Scale bar: 5μm. Data was representative of 3 independent experiments and analyzed using one-way ANOVA. *P < 0.05, **P < 0.01, ***P < 0.001, ****P < 0.0001

ER-mitochondrial contact sites are known to orchestrate many important cellular functions, including calcium signaling, mitochondrial function, and ER stress, which are known to be dysregulated in AD [31]. The expression of Sig-1r was known to be critical for ER-mitochondrial contacts since the Sig-1r activation regulate calcium transport from ER to mitochondria and indirectly regulate TCA cycles [44, 45]. Herein, the effects of DMT on the ER-mitochondria distance was examined in hippocampal neurons of 3×TG-AD and Sig-1r knockdown mice. Quantitative transmission electron microscope analysis displayed a significant increase in the ER-mitochondrial distance of 3×TG-AD mice relative to WT mice, whereas chronic DMT shortened the ER-mitochondrial distance to the value of WT mice (Fig. 3A, B). Sig-1r knockdown mice had an approximately 40% wider distance than controlled mice. DMT completely rescued the distance between the two organelles (Fig. 3C, D**)**.

To further detect effects of DMT on ER-mitochondrial physical contact, cultured neurons were treated with 20 μM Aβ_25-35_ plaque and 1 μM BD1063 with or without DMT treatment for 24 h. The contact points between the ER and mitochondria were verified using PLA technology. The PLA dots was used to measure the proximity of IP3R3-VDAC1 pair, indicating the contact points between the ER and mitochondrial, as the general distance between the two organelles is 20-40 nm and PLA can detect protein pairs with less than 40 nm away from each other [46]. A striking decrease in PLA dots was observed in the Aβ-exposed cells relative to the normal cells. The presence of DMT greatly increased the number of PLA dots in the cells exposed to Aβ, but did not show great effects in the control cells (Fig. 3E, F). The number of PLA dots markedly decreased in the cells exposed to BD1063. BD1063 co-treatment with DMT increased the number of PLA dots (Fig. 3G, H). These results suggested that DMT improved the ER-mitochondrial crosstalk under the pathological conditions.

### DMT modulated the expression of MAM-associated proteins in mice brain

**Fig. 4:**
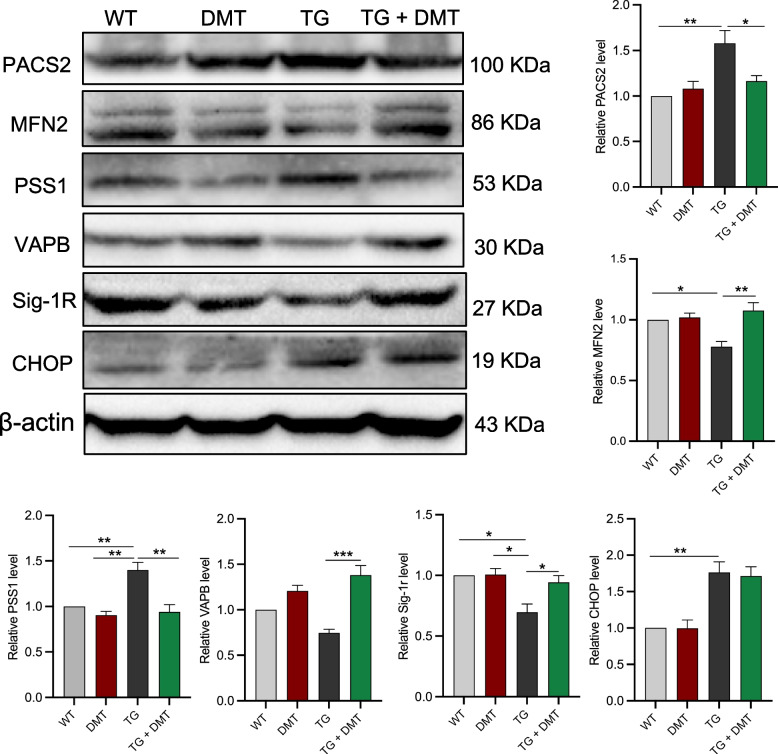
DMT modulates the expression of MAM-associated proteins in 3×TG-AD mice brains. Western blot was used to determine the expression of MAM-associated proteins. Data were analyzed using one-way ANOVA (*n* = 3 per group). *P < 0.05, **P < 0.01, ***P < 0.001

In addition to IP3R and VDAC1 regulating calcium transfer from ER to mitochondria, other MAM-associated proteins, such as PACS2, PSS1, MFN2, Sig-1r, VAPB, and CHOP, also directly mediate ER-mitochondrial contact and calcium homeostasis [30–32, 47]. Give that DMT significantly modulated ER-mitochondrial contact in neuronal tissues, we further evaluated the effects of DMT on the expression of these MAM-associated proteins in the whole brain of mice using Western blot (Fig. 4).

The expression levels of PACS2, PSS1, and CHOP in the 3×TG-AD mice were much higher than those of WT mice, DMT markedly suppressed the higher levels of PACS2 and PSS1 (P ≤ 0.0339), but did not show significant effects on CHOP expression. The expression of the remaining three proteins, MFN2, Sig-1r, and VAPB, markedly decreased in 3×TG-AD mice relative to WT mice (P ≤ 0.0390). DMT remarkably restored the levels of the three proteins (P ≤ 0.0216). The level of these 6 MAM-associated proteins did not show significant changes in the WT mice treated with DMT.

### DMT modulated Ca^2+^ dynamic between ER and mitochondria

**Fig. 5:**
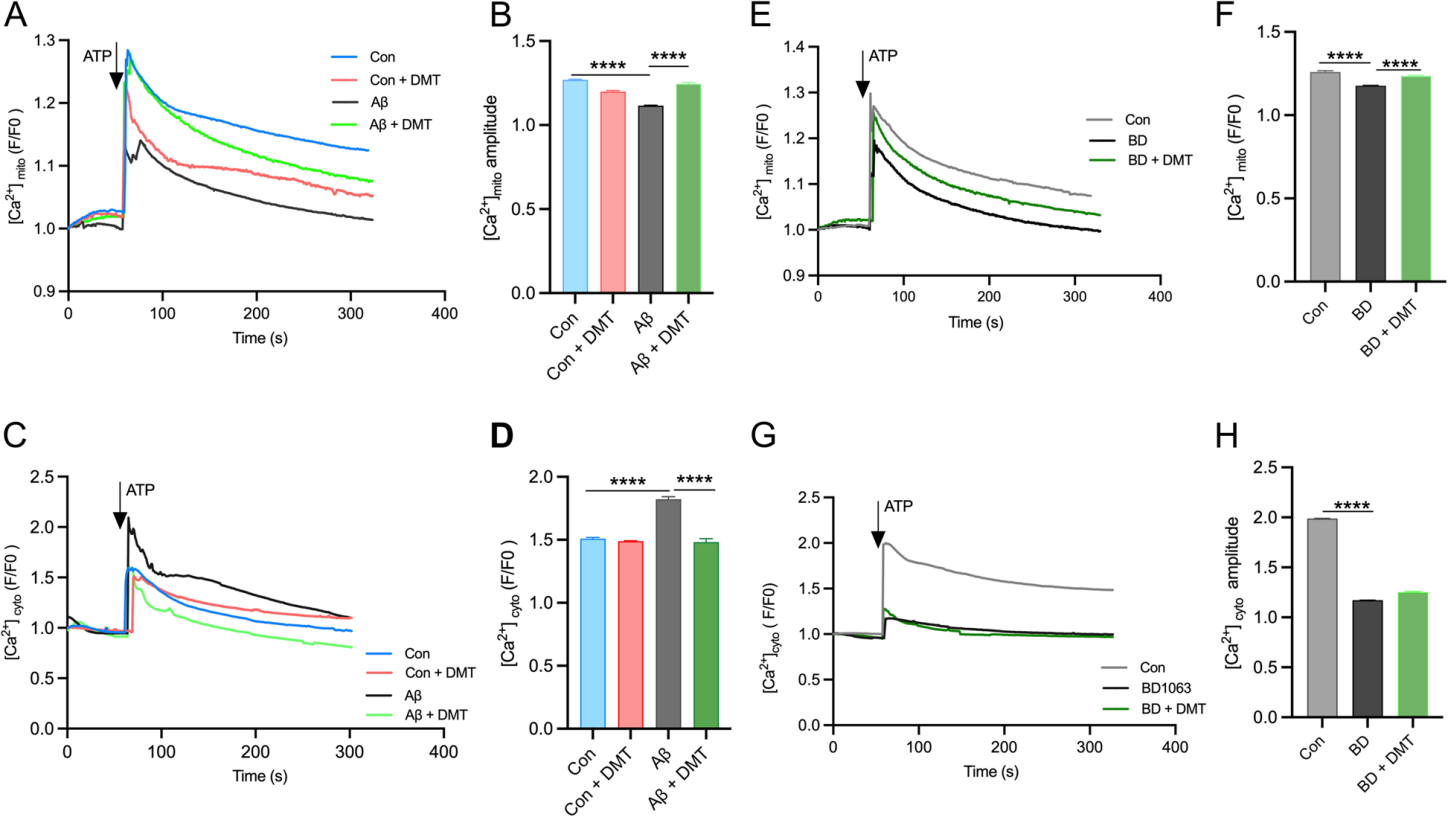
DMT modulates the calcium transfer between ER and mitochondria. Cytosolic and mitochondrial Ca^2+^ were measured in the primary hippocampus by using live cell calcium imaging. **A**, **B** and **C**, **D** show the mitochondrial and cytosolic calcium, respectively, in the cells exposed to Aβ with or without DMT treatment. **E**, **F** and **G**, **H** show the mitochondrial and cytosolic calcium, respectively, in the cells exposed to BD1063 with or without DMT treatment. **A**, **C**, **E**, **G** show the representative figure of Ca^2+^ peaks after ATP treatment. **B**, **D**, **F**, **H** show the the amplitude peak of calcium levels after ATP treatment. Data were obtained from 3 independent experiments and analyzed using two-way ANOVA. [Ca^2+^]_mito_, mitochondrial calcium; [Ca^2+^]_cyto_, cytosolic calcium. BD, BD1063. ****P < 0.0001

After evaluating the effect of DMT on the main factors impacting on the Ca^2+^ transport, ER-mitochondrial contacts and MAM-associated proteins, we further determined effects of DMT on the Ca^2+^ transfer efficiency between ER and mitochondria. Ca^2+^ uptake in the organelles was assessed in the presence and absence of DMT with and without co-treatment with BD1063 and Aβ in primary hippocampal neurons. Following 24 hours of treatment, DMT had no effects on mitochondrial and cytosolic Ca^2+^ response to ATP stimulation (Fig. 5A-D). The Aβ exposure evoked an approximate 15% decrease and 24% increase of Ca^2+^ amplitude in mitochondria and cytosol, respectively. This result was consistent with previous studies, in which the decrease in mitochondrial Ca^2+^ in the Aβ -treated cells was considered to be the loss of MMP that significantly reduced mitochondrial Ca^2+^ uptake capacity [48]. Co-administration with DMT almost entirely reversed the Aβ-evoked alteration of Ca^2+^ amplitude in the two subcellular components (Fig. 5A-D). BD1063 markedly also lowered mitochondrial Ca^2+^ level. The cytosolic Ca^2+^ amplitude of the BD1063-treated cells was only 59.1% of the controlled cells. Co-treatment with DMT restored mitochondrial Ca^2+^ amplitude to the control value, but did not change the cytosolic level (Fig. 5E-H).

### DMT protected mitochondrial function in the primary hippocampal neurons

**Fig. 6:**
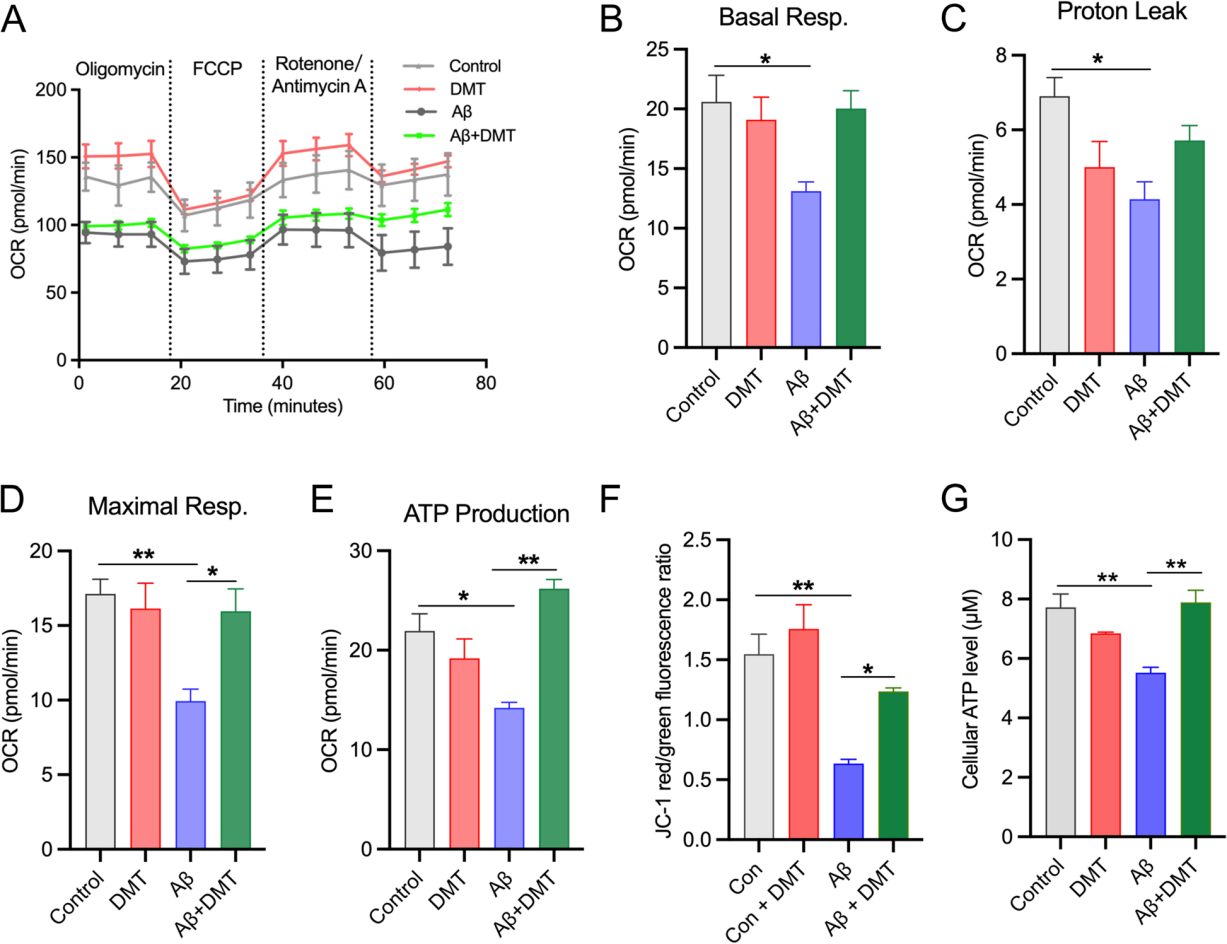
DMT rescues mitochondrial dysfunction in the primary hippocampal neurons. **A** Oxygen consumption rate (OCR) at baseline and following: oligomycin, FCCP, and rotenone + antimycin A. **B**, **C**, **D**, **E** show the quantification of basal respiration, proton leak, maximal respiration, and ATP production. **F** shows the quantification of the mitochondrial membrane potential (MMP). **G** shows the quantification of the cellular ATP level. Data were obtained from 3 independent experiments and analyzed using two-way ANOVA. *P < 0.05, **P < 0.01

Having established that DMT was sufficient to maintain mitochondrial Ca^2+^ homeostasis, we next considered the effect of DMT on the mitochondrial Ca^2+^ -linked cellular process, including oxidative phosphorylation (OXPHOS) and ATP synthase, in the in vitro model of AD. Aβ-treated cells were monitored for changes in OXPHOS by measuring mitochondrial oxygen consumption rates (OCR) in a seahorse assay. The Aβ-exposed cells also had strikingly lower OCR values on basal respiration, proton leak, maximal respiratory capacity, and ATP-linked respiration (Fig.6A-E). Correspondingly, MMP and cellular ATP levels also decreased (Fig. 6F-G). This result was expected because it has been known that maintaining the TCA cycle requires sufficient flux of Ca^2+^ from the ER to mitochondria [49]. Therefore, the loss of mitochondrial Ca^2+^ in the cells exposed to Aβ may impair OXPHOS activity, leading to mitochondrial damage, collapse of MMP, and the decrease of ATP synthase. The aforesaid parameters, maximal respiratory capacity and ATP-linked respiration, were rescued post DMT treatment (Fig.6D, E). DMT treatment in control cells had no effect on OCR. DMT and Aβ co-incubation markedly reversed intracellular ATP levels and MMP compared to incubation with Aβ alone (P < 0.050) (Fig. 6F, G).

## Discussion

The current study represents a systematic investigation on the anti-AD effects of DMT and its association with the modulation of neuronal Sig-1r-mediated ER-mitochondria signaling in the 3×TG-AD transgenic mouse model of AD and Aβ-exposed primary hippocampal neurons with additional utilization of Sig-1r knockdown mice and the Sig-1r antagonist BD1063.

This study revealed robust effects of DMT in mitigating spatial memory impairment and Aβ deposits in the transgenic AD model mice. In the water maze test, following 3 weeks of the treatment, DMT markedly shortened the latency to the platform in multiple days of training phase, and increased time and number of entries into the platform in the probe trial of the model mice. In parallel, chronic DMT largely reduced Aβ plaques in the hippocampus and PFC, the two brain regions closely associated with encoding, retrieval, and consolidation of memories [50]. One previous study has shown that chronic DMT (2 mg/kg for 21 days) also improved learning and memory in water maze and novel object recognition test in intact mice; the combination with ritanserin, a 5-HT_2_ receptor antagonist, however, did not abolish the behavioral effects of DMT [21]. DMT promoted adult neurogenesis of the hippocampus via Sig-1r, but not 5-HT_1A_ and 5-HT_2A_ receptors [21]. These results suggest that the anti-AD effects of DMT observed in this study is more closely associated with its modulation of Sig-1r than 5-HT receptor systems.

Subsequently, we further examined the role of Sig-1r in DMT’s effects. While a marked decreased level of Sig-1r in the hippocampus of 3×TG-AD and Sig-1r knockdown mice was observed using immunofluorescent detection, chronic DMT consistently and entirely returned the expression of Sig-1r to the control levels in the 3×TG-AD mice. These results proved that DMT as agonist could protect brain Sig-1r activity from AD pathological insults. It also indicated an involvement of the decreased Sig-1r function in the neuropathology of AD, which is consistent with previous studies, demonstrating a large decrease and loss of Sig-1r in brain regions of individuals in early stage of AD [5, 51]. In other studies, it was found that another Sig-1r agonist, PRE084, improved mitochondrial respiratory impairment and alleviated blood-brain barrier disruption in the Aβ-injected animals [14, 52]. Both PRE084 and DMT significantly reduced Aβ-induced neuroinflammatio n[42]. Compared with DMT, PRE084 significantly improved hippocampal neurogenesis in the Aβ-injected mice, which may be due to PRE084’s higher affinity for Sig-1r relative to DMT [42]. However, whether there exit differences between PRE084 and DMT in improving others AD pathology remains for further evaluation, as DMT is an agonist acting at multiple receptors such as Sig-1r, 5-HT2, and TAAR, while PRE084 is a highly selective Sig-1r agonist [23, 42].

Given that ER-mitochondria interface with MAMs is a key site where Sig-1r mediates calcium signaling between the two organelles [6], we hence examined the effects of DMT on the integrity of ER-mitochondria physical contacts. Both transgenic and Sig-1r knockdown mice displayed markedly wider gaps at ER and mitochondria contact points as compared to wild type mice. Similarly, following the exposure of cultured hippocampal neurons to Aβ oligomers and BD1063 for 24 hours, number of PLA-labeled dots indicative of ≤ 40 nm contact clefts between the two organelles strikingly decreased, proving the assertion that the impaired ER-mitochondria integrity is a universal and probably early event occurred in the pathogenesis of AD [4]. Co-administration with DMT, however, largely and even completely recuperated the two organelles’ contact gaps in both in vivo and intro paradigms. It appeared that the protective effects of DMT against the AD-related impairment of the subcellular integrity were mainly derived from its activation of Sig-1r.

This study further revealed that the model mice had apparently abnormal expression of the six MAM-associated proteins examined, manifesting as upregulated expression of PACS2, PSS1 and CHOP, and downregulated expression of MFN2, VAPB and Sig-1r. The first three proteins are widely involved in free radical production and ER stress-induced apoptosis, while they are key components of MAMs in regulating calcium transfer [3]. The last three proteins play a critical role in the maintenance and operation of mitochondrial network in particular ER-mitochondrial communication [3]. One previous study also has shown the upregulated expression of PACS2 and PSS1 in multiple brain regions of AD mouse model and in cortical tissues of AD patients; and the upregulation occurred prior to the appearance of Aβ plaques [5]. It is suggested that the dysregulation of MAM-associated proteins may represent an early aberrant event of ER-mitochondrial interplay that perhaps triggers AD pathological cascades. However, chronic DMT partially and even entirely reversed the expression of all the MAM-associated proteins to the levels of WT control mice, except for CHOP, indicating an association of DMT’s effects in preventing ER-mitochondria interplay with its modulation of MAM-associated proteins. The increased CHOP has been suggested in the progression of AD by sensitizing neuronal cells to apoptosis [53]. Other two MAM-associated proteins, PACS-2 and PSS1, are involved in modulating lipid synthesis of the ER. PACS-2 also could integrate ER-mitochondria communication and apoptosis [54, 55]. This study revealed that DMT had significant effects in suppressing the increased expression of PACS2 and PSS1, but not CHOP. It seems that DMT may particularly restore lipid synthesis of the ER, but had less effects in modulating cell apoptosis. 

Dysregulation of MAM-mediated calcium homeostasis has been implicated in the neuropathology of AD and other neurodegenerative diseases [56]. In this study, we examined the effects of DMT on neuronal calcium dynamics by measuring mitochondrial and cytosolic calcium amplitude under DMT co-treatment with Aβ and BD1063. While the Aβ exposure for 24 hours induced a decrease of mitochondrial and an elevation of cytosolic calcium amplitude, BD1063 reduced both compartment calcium amplitudes. It is likely that the neurotoxin and the Sig-1r antagonist may target subcellular compartments differentially, i.e., Aβ mainly impaired mitochondrial calcium dynamics by inhibiting the uptake, in turn, causing the accumulation of the ion in the cytosol. It might be plausible as mitochondria among organelles is the most vulnerable to oxidative and neurotoxic insults [57]. Whereas BD1063 probably suppressed calcium influx and storage via the blockade of ER’s Sig-1r. While DMT co-treatment overturned mitochondrial calcium uptake in the two types of pathological insults and Aβ-evoked decrease of cytosolic calcium level, it did not affect the BD1063-induced suppression of cytosolic calcium level. Similar results were also observed in a previous study, revealing that mitochondrial, but not cytosolic, calcium uptake was markedly increased following SA4503, an agonist of Sig-1r, co-treatment with Aβ or tunicamycin, a neurotoxin, in SH-SY5Y cells, a cell model of neurodegenerative disorders [5]. It thus appeared that the effects of DMT in protecting neuronal calcium homeostasis may be principally achieved by restoring Sig-1r-mediated MAM calcium mechanisms.

We also noted that DMT improved the expression of Sig-1r in the AD models. It is possible that DMT helps to compensate for the loss of signalling pathways and other receptors. The Sig-1r itself has been found to have pleiotropic neuroprotective effects, such as shuttling calcium from the ER to mitochondria to prevent mitochondrial stress, prevent apoptosis, and induce cell proliferation and growth [41]. Targeting Sig-1r in disease states may therefore have multiple neuroprotective benefits. Herein, our results indicated that DMT regulated calcium transfer between and mitochondria to restore mitochondrial function via Sig-1r activation. Also, DMT effectively repaired mitochondrial function manifesting as the improvement in the mitochondrial OXPHOS capacity and ATP synthesis in the AD models.

DMT has been found to enhance cognitive performance in adult mice probably via Sig-1r modulation of neurogenesis [21], confirming, once again, the crucial function of Sig-1r in the DMT’s nootropic effects. It also could, at least in part, explain DMT as an entheogenic beverage often used to enhance perception, mood, consciousness, cognition, and behavior in ritual and recreational activities. Furthermore, as the endogenous ligand, DMT could exert the behavioural effect by binding to Sig-1r, which is abundant in the brain [19]. This study elucidated the underlying intracellular molecular mechanism that DMT improved cognitive dysfunction in AD via activation of Sig-1r. Additionally, DMT is also the agonist of TAAR and 5-HT receptors, which are regarded as targets for the treatment of AD [58, 59]. Therefore, DMT may exert anti-AD effect through multiple receptors-mediated signalling. Previous studies have discussed the importance of targeting multiple pathways in combating the neurodegenerative disease AD, particularly using drugs that target multiple receptors [60]. Therefore, DMT is a potentially important drug for the treatment of AD.

There are multiple limitations that should be taken into consideration. Firstly, this study only delineated the neuronal mechanisms, but did not detect the role of glia in the nootropic effects of DMT. Sig-1r is also expressed in microglia and it is involved in the protective effects of DMT against brain ischemia [61]. Whether and to what extent microglial Sig-1r is involved in the anti-AD effects of DMT deserve for further estimation. Secondly, although the previous study has revealed that the nootropic effects of DMT were less associated with 5-HT receptor systems than Sig-1r [21], whether there exist synchronic, additive, or even synergistic effects of the two receptor systems should be an intriguing question for the future study of DMT. Finally, although, unlike most hallucinogenic drugs, DMT did not cause or caused less tolerance, dependence, and withdrawal symptoms for acute and short-term use [23], the potential side effects of the long-term use should be carefully examined. Additionally, DMT should have acceptable safety parameters for use due to its high vulnerability to abuse. It also should be pay attention to whether long-term effects of DMT use is associated with Hallucinogen Persistence Perception Disorder.

## Conclusion

**Fig. 7:**
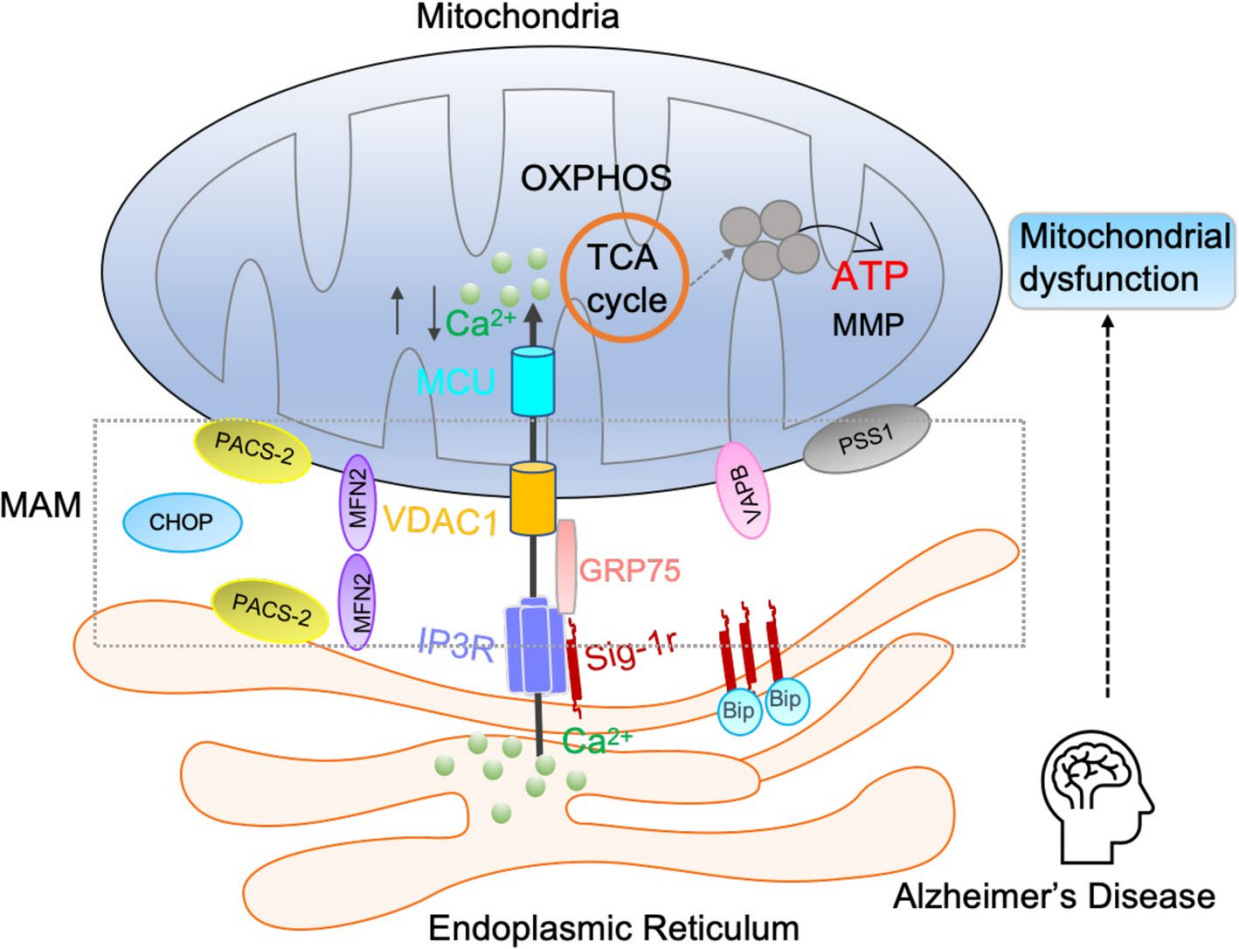
The putative mechanism of DMT improving the spatial learning and memory in animal models of AD. The current study demonstrated that the anti-AD effects of DMT are associated with its restoration of neuronal ER-mitochondria crosstalk via the Sig-1r activation. DMT modulates the mitochondrial calcium uptake and ER-mitochondrial contacts in the in vivo and in vitro models, facilitates the TCA cycle, and protects against mitochondrial dysfunction in Alzheimer’ disease

Taken together, the current study demonstrated that the anti-AD effects of DMT are associated with its restoration of neuronal ER-mitochondria crosstalk via the Sig-1r activation (Fig. 7). Sig-1r was identified as a target for maintenance of mitochondrial calcium homeostasis and restoration of mitochondrial function, particularly mitochondrial calcium uptake. It then improved mitochondrial OXPHOS capacity and ATP production. DMT could serve a novel preventive and therapeutic agent against AD.

### Supplementary Information

**Supplementary Material 1.**

## Data Availability

The datasets used and/or analyzed during the current study are available from the corresponding author on reasonable request.

## Abbreviations

Aβ: β-amyloid peptide
AD: Alzheimer’s disease
DMT: N, N-dimethyltryptamine
EGF: Epidermal growth factor
ER: Endoplasmic reticulum
FGF: Fibroblast growth factor
GC/MS: Chromatography–mass spectrometry
DG: Dentate gyrus
HBSS: Hank’s balanced salt solution
MAM: Mitochondria-associated endoplasmic reticulum membrane
OXPHOS: Oxidative phosphorylation
PFA: Paraformaldehyde
PVDF: Polyvinylidene difluoride
RIPA: Radioimmunoprecipitation assay
SDS-PAGE: Sodium dodecyl sulfate-polyacrylamide gel electrophoresis
Sig-1r: Sigma-1 receptor
VDAC1: Voltage dependent anion channel

## References

[1] Galvin JE (2017). The social and economic burden of frontotemporal degeneration. Neurol..

[2] Athar T, Al Balushi K, Khan SA (2021). Recent advances on drug development and emerging therapeutic agents for Alzheimer’s disease. Molecular biol rep..

[3] Eysert F (2020). Molecular dysfunctions of mitochondria-associated membranes (MAMs) in Alzheimer’s disease. Int J Molecular Sci..

[4] Markovinovic A (2022). Endoplasmic reticulum–mitochondria signaling in neurons and neurodegenerative diseases. J Cell Sci..

[5] Hedskog L (2013). Modulation of the endoplasmic reticulum–mitochondria interface in Alzheimer’s disease and related models. Proceed Nat Academ Sci..

[6] Vance, J.E., MAM (mitochondria-associated membranes) in mammalian cells: lipids and beyond. Biochimica et Biophysica Acta (BBA)-Molecular Cell Biol Lipids., 2014. 1841(4): 595-609. 10.1016/j.bbalip.2013.11.014 24316057

[7] Tsai S-YA (2014). Sigma-1 receptor chaperones in neurodegenerative and psychiatric disorders. Expert opin therapeut targets..

[8] Qin J (2019). Activation of sigma-1 receptor by cutamesine attenuates neuronal apoptosis by inhibiting endoplasmic reticulum stress and mitochondrial dysfunction in a rat model of asphyxia cardiac arrest. Shock..

[9] Ke M (2022). Sigma-1 receptor overexpression promotes proliferation and ameliorates cell apoptosis in β-cells. Molecular Med Rep..

[10] Hayashi T, Su T-P (2007). Sigma-1 receptor chaperones at the ER-mitochondrion interface regulate Ca2+ signaling and cell survival. Cell..

[11] Lan Y (2019). Novel radioligands for imaging sigma-1 receptor in brain using positron emission tomography (PET). Acta Pharmaceutica Sinica B..

[12] Leitner ML (1994). Regional variation in the ratio of σ1 to σ2 binding in rat brain. Europ J Pharmacol..

[13] Alonso G (2000). Immunocytochemical localization of the sigma1 receptor in the adult rat central nervous system. Neurosci..

[14] An Y (2022). Activation of the sigma-1 receptor attenuates blood–brain barrier disruption by inhibiting amyloid deposition in Alzheimer’s disease mice. Neurosci Lett..

[15] Ryskamp DA, et al. Neuronal sigma-1 receptors: signaling functions and protective roles in neurodegenerative diseases. Front neurosci. 2019;13:862. 10.3389/fnins.2019.00862 PMC6736580 31551669

[16] Martín-Guerrero SM, et al. Targeting ER-mitochondria signaling as a therapeutic target for frontotemporal dementia and related amyotrophic lateral sclerosis. Front Cell Develop Biol. 2022;10:915931. 10.3389/fcell.2022.915931 PMC9184680 35693938

[17] Barker SA (2022). Administration of N, N-dimethyltryptamine (DMT) in psychedelic therapeutics and research and the study of endogenous DMT. Psychopharmacol..

[18] Hamill J (2019). Ayahuasca: psychological and physiologic effects, pharmacology and potential uses in addiction and mental illness. Curr neuropharmacol..

[19] Fontanilla D (2009). The hallucinogen N, N-dimethyltryptamine (DMT) is an endogenous sigma-1 receptor regulator. Science..

[20] Szabo A (2016). The endogenous hallucinogen and trace amine N, N-dimethyltryptamine (DMT) displays potent protective effects against hypoxia via sigma-1 receptor activation in human primary iPSC-derived cortical neurons and microglia-like immune cells. Front neurosci..

[21] Morales-Garcia JA (2020). N, N-dimethyltryptamine compound found in the hallucinogenic tea ayahuasca, regulates adult neurogenesis in vitro and in vivo. Translational Psychiatry..

[22] D’Souza DC (2022). Exploratory study of the dose-related safety, tolerability, and efficacy of dimethyltryptamine (DMT) in healthy volunteers and major depressive disorder. Neuropsychopharmacol..

[23] Barker SA (2018). N, N-dimethyltryptamine (DMT), an endogenous hallucinogen: past, present, and future research to determine its role and function. Front neurosci..

[24] Sterniczuk R (2010). Characterization of the 3xTg-AD mouse model of Alzheimer's disease: part 2*.* Behavioral and cognitive changes. Brain res..

[25] Cheng D (2021). Tortoise Plastron and Deer Antler Gelatin Prevents Against Neuronal Mitochondrial Dysfunction In Vitro: Implication for a Potential Therapy of Alzheimer’s Disease. Front pharmacol..

[26] Morris RG (1982). Place navigation impaired in rats with hippocampal lesions. Nature..

[27] Cheng D (2023). Minocycline, a classic antibiotic, exerts psychotropic effects by normalizing microglial neuroinflammation-evoked tryptophan-kynurenine pathway dysregulation in chronically stressed male mice. Brain Behav Immun..

[28] Rajamohamedsait, H.B. and E.M. Sigurdsson, Histological staining of amyloid and pre-amyloid peptides and proteins in mouse tissue. Amyloid Proteins: Methods and Protocols. 2012;849:411–424. 10.1007/978-1-61779-551-0_28 PMC3859432 22528106

[29] Lam J (2021). A universal approach to analyzing transmission electron microscopy with ImageJ. Cells..

[30] Wang Y (2015). PERK/CHOP contributes to the CGK733-induced vesicular calcium sequestration which is accompanied by non-apoptotic cell death. Oncotarget..

[31] Wilson EL, Metzakopian E (2021). ER-mitochondria contact sites in neurodegeneration: genetic screening approaches to investigate novel disease mechanisms. Cell Death Different..

[32] Krols M (2016). Mitochondria-associated membranes as hubs for neurodegeneration. Acta neuropathol..

[33] Wieckowski MR (2009). Isolation of mitochondria-associated membranes and mitochondria from animal tissues and cells. Nature protocols..

[34] Cozzolino M (2004). Apoptosome inactivation rescues proneural and neural cells from neurodegeneration. Cell Death Different..

[35] Tong BC-K (2022). Lysosomal TPCN (two pore segment channel) inhibition ameliorates beta-amyloid pathology and mitigates memory impairment in Alzheimer disease. Autophagy..

[36] D’Eletto M (2018). Transglutaminase type 2 regulates ER-mitochondria contact sites by interacting with GRP75. Cell rep..

[37] Jadiya P (2019). Impaired mitochondrial calcium efflux contributes to disease progression in models of Alzheimer’s disease. Nature commun..

[38] Li H (2014). Imaging of mitochondrial Ca2+ dynamics in astrocytes using cell-specific mitochondria-targeted GCaMP5G/6s: mitochondrial Ca2+ uptake and cytosolic Ca2+ availability via the endoplasmic reticulum store. Cell calcium..

[39] Zorova LD (2018). Mitochondrial membrane potential. Analyt biochem..

[40] Toyohara, J., M. Sakata, and K. Ishiwata, Imaging of sigma1 receptors in the human brain using PET and [11C] SA4503. Central Nervous System Agents in Medicinal Chemistry (Formerly Current Medicinal Chemistry-Central Nervous System Agents), 2009. 9(3): 190-196. 10.2174/1871524910909030190 20021353

[41] Prasanth MI (2021). The emerging role of the sigma-1 receptor in autophagy: Hand-in-hand targets for the treatment of Alzheimer’s. Expert Opin Therapeut Targets..

[42] Borbély E (2022). Impact of two neuronal Sigma-1 receptor modulators, PRE084 and DMT, on neurogenesis and neuroinflammation in an Aβ1–42-injected, wild-type mouse model of AD. Int J Molecular Sci..

[43] Omi T (2014). Fluvoxamine alleviates ER stress via induction of Sigma-1 receptor. Cell death disease..

[44] Weng T-Y, Tsai S-YA, Su T-P (2017). Roles of sigma-1 receptors on mitochondrial functions relevant to neurodegenerative diseases. J biomed sci..

[45] Wu NH (2021). Emerging benefits: pathophysiological functions and target drugs of the sigma-1 receptor in neurodegenerative diseases. Molecular Neurobiol..

[46] Csordás G (2006). Structural and functional features and significance of the physical linkage between ER and mitochondria. J cell biol..

[47] Jiang R-Q, Li Q-Q, Sheng R. Mitochondria associated ER membrane and cerebral ischemia: molecular mechanisms and therapeutic strategies. Pharmacol Res. 2023;191:106761. 10.1016/j.phrs.2023.106761 37028777

[48] Calvo-Rodriguez M (2019). Amyloid β oligomers increase ER-mitochondria Ca2+ cross talk in young hippocampal neurons and exacerbate aging-induced intracellular Ca2+ remodeling. Front cell neurosci..

[49] Cardenas C (2010). Essential regulation of cell bioenergetics by constitutive InsP3 receptor Ca2+ transfer to mitochondria. Cell..

[50] Preston AR, Eichenbaum H (2013). Interplay of hippocampus and prefrontal cortex in memory. Curr biol..

[51] Jin J-L (2015). Roles of sigma-1 receptors in Alzheimer’s disease. Int J clin experiment med..

[52] Lahmy V (2015). Mitochondrial protection by the mixed muscarinic/σ1 ligand ANAVEX2-73, a tetrahydrofuran derivative, in Aβ25–35 peptide-injected mice, a nontransgenic Alzheimer’s disease model. Front cell neurosci..

[53] Iadecola C (1997). Delayed reduction of ischemic brain injury and neurological deficits in mice lacking the inducible nitric oxide synthase gene. J Neurosci..

[54] Simmen T (2005). PACS-2 controls endoplasmic reticulum–mitochondria communication and Bid-mediated apoptosis. EMBO J..

[55] Xu L, Wang X, Tong C (2020). Endoplasmic reticulum–mitochondria contact sites and neurodegeneration. Front Cell Develop Biol..

[56] Lim D (2021). Ca2+ handling at the mitochondria-ER contact sites in neurodegeneration. Cell Calcium..

[57] Prentice H, Modi JP, Wu J-Y. Mechanisms of neuronal protection against excitotoxicity, endoplasmic reticulum stress, and mitochondrial dysfunction in stroke and neurodegenerative diseases. Oxidat med cell longevity. 2015;964518. 10.1155/2015/964518 PMC4630664 26576229

[58] Leo D (2022). Trace Amine Associate Receptor 1 (TAAR1) as a New Target for the Treatment of Cognitive Dysfunction in Alzheimer’s Disease. Int J Molecular Sci..

[59] Reyna NC, et al. Anxiety and Alzheimer’s disease pathogenesis: focus on 5-HT and CRF systems in 3xTg-AD and TgF344-AD animal models. Fronti Aging Neurosci. 2023;15:1251075. 10.3389/fnagi.2023.1251075 PMC10699143 38076543

[60] Gong C-X (2022). Multi-targets: An unconventional drug development strategy for Alzheimer’s disease. Front Aging Neurosci..

[61] Nardai S (2020). N, N-dimethyltryptamine reduces infarct size and improves functional recovery following transient focal brain ischemia in rats. Experiment Neurol..

