# Supplementary material for: N, N-Dimethyltryptamine, a natural hallucinogen, ameliorates Alzheimer’s disease by restoring neuronal Sigma-1 receptor-mediated endoplasmic reticulum-mitochondria crosstalk
Source: Alzheimers Res Ther. 2024 May 1;16:95. doi: 10.1186/s13195-024-01462-3 (PMC11061967; doi:10.1186/s13195-024-01462-3)

**Supplementary material**

**1. Method**

**Cell viability assay**

Cell viability was evaluated using a Cell Counting Kit-8 (CCK-8) assay (ApeBio, USA). Briefly, cells were seeded into 96-well plates at a density of 8× 10^3^ cells/well and incubated for 7 days. Then cells were treated with various agents according to the purpose of the study. To determine the optimal DMT concentration that significantly improves cell viability, cells were treated for 24 h with DMT concentrations ranging from 0.04 to 125 μM. To evaluate the effect of DMT on the cell viability under Aβ and BD1063 conditions, cells were treated with Aβ_25-35_ and BD1063 at 20 μM and 1 μM final concentration, respectively, in the presence or absence of DMT. After the completion of treatment, 10 μL of CCK-8 solution was added to each well and incubated for 2 hours. The absorbance of each well at 450 nm was measured on a microplate reader (Bio-Rad, USA).

**Analysis of Sig-1r level in the 3×TG-AD mice brain with the disease progression**

The expression of Sig-1r was examined using Western blot in the whole brain (cerebellum excluded). The purified mitochondria-associated membranes (MAM) was lysed in RIPA buffer plus the protease inhibitor. 30 μg of total proteins from the MAM were separated by 10% sodium dodecyl sulfate-polyacrylamide gel electrophoresis (SDS-PAGE) and transferred to polyvinylidene difluoride (PVDF) membranes following protein determination. The membranes were then incubated with 5% BSA for 1.5 hours, followed by the primary antibodies against Sig-1r (Sant Cruz, 1:1000) incubated for 24 hours. The secondary antibodies were then incubated for 1.5 hours. The quantitative expression level of proteins was determined using the ChemiDoc XRS+ imaging system (Bio-Rad, USA) after being detected with an ECL select detection reagent (GE Healthcare, USA).

**2. Results**

**2.1 DMT promoted neural cell viability in the cells exposed to Aβ_25–35._**

Regarding the cell viability when primary hippocampal cells were exposed to DMT, with a range of concentrations from 0.04 to 125 μM were assessed. DMT stimulated cell growth within the range of 0.04-25μM, with maximal promotion at 1 and 5 μM (P ≤ 0.0446) (Fig. S1A). Herein, 1 μM of DMT was selected for the subsequent in vitro study.


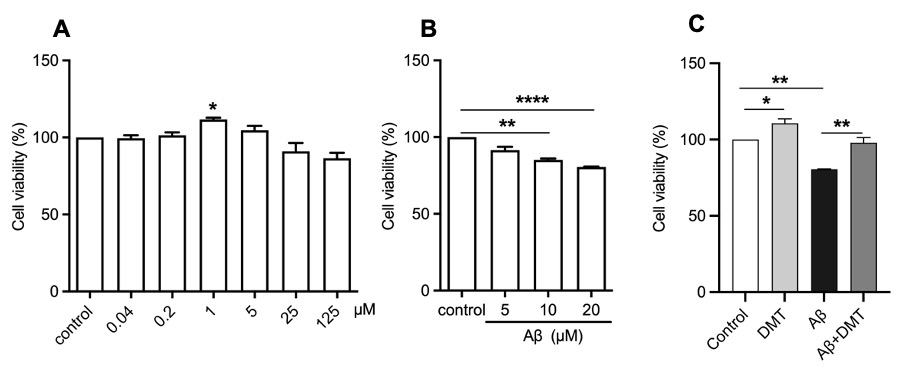
Further study was performed to characterize the role of DMT in protecting cell viability under Aβ_25–35_ condition. Firstly, primary hippocampal neurons were exposed to Aβ_25–35_ for 24 h at the concentrations ranging from 5-20 μM. The results showed that 20 μM Aβ_25–35_ significantly reduced cell viability to 80.60% (P = 0.0012), which was used in the subsequent in vitro studies (Fig. S1B). Then Cells were treated with 1 μM DMT in the presence or absence of 20 μM Aβ_25–35_ for 24h. The results showed that 20 μM Aβ_25-35_ significantly reduced cell viability to 80.6% (P = 0.0012). DMT treatment remarkably increased cell viability of cells exposed to Aβ_25–35_ (P = 0.0024) (Fig. S1C).

**Fig. S1** DMT improves neuronal cell viability and protects cells against Aβ_25-35_ stress. (A) The effect of DMT on the cell viability of primary hippocampal cells. (B) Cell viability in primary hippocampal cell exposed to varying Aβ_25-35_ concentrations_._ (C) The improvement of cell viability upon DMT treatment with or without Aβ_25-35_ stress in the primary hippocampal neurons. Data was expressed as mean ± SEM and analyzed using one-way ANOVA, followed by Tukey post hoc test. *P ＜ 0.05, **P ＜ 0.01, ****P ＜ 0.0001.

**2.2 DMT promoted neural cell viability in the cells exposed to BD1063**

To demonstrate the modulation effect of DMT on Sig-1r in the in vitro study, primary hippocampal cells were treated with the antagonist of Sig-1r, BD1063, in the presence or absence of DMT. Results showed that Sig-1r inhibition with BD1063 substantially decreased cell viability, whereas DMT treatment increased cell viability in BD1063-exposed cell (P = 0.0007) (Fig. S2).

**
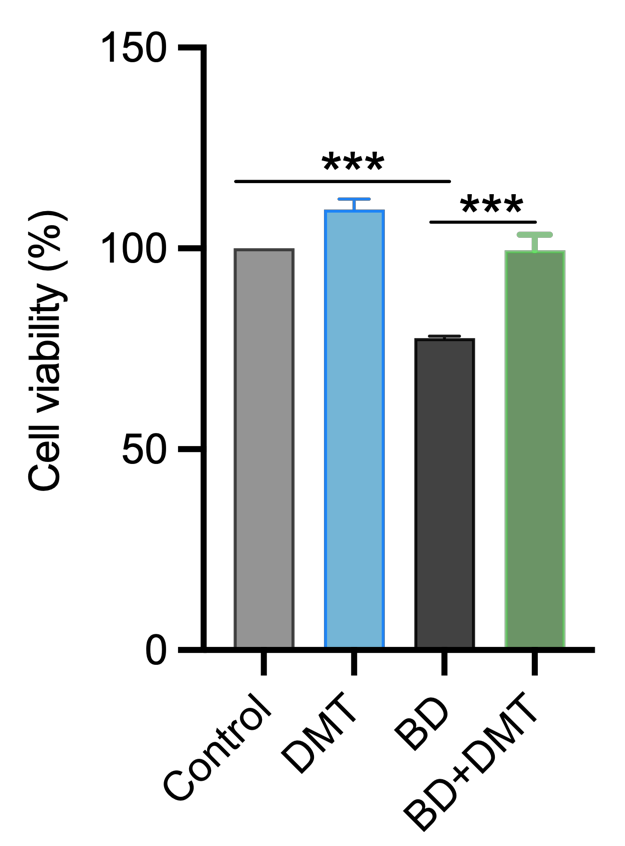
**

**Fig. S2** DMT improves cell viability in the cells exposed to BD1063. Cells exposed to the Sig-1r antagonist, BD1063, in the presence or absence of DMT. Data was expressed as mean ± SEM and analyzed using one-way ANOVA, followed by Tukey post hoc test. BD, BD1063. *P ＜ 0.05, **P ＜ 0.01, ****P* ＜ 0.001.

**2.3 Sig-1r in the hippocampus of 3×TG-AD mice significantly decreased with the progression of AD**

The expression of Sig-1r in 3×TG-AD mice was investigated with the disease progression. For this purpose, 3-month-old (before Aβ deposition), 6-month-old (apparent Aβ deposition) and 12-month-old (sever Aβ deposition) mice were used for western blot analysis according to their disease progression of Aβ pathology [1], and the match-aged WT mice served as control. As shown in Fig. S3, Sig-1r expression levels in 3×TG-AD mice were higher than WT mice before or on apparent Aβ deposition (P≤ 0.0042), but lower than WT mice at 12- month-old of the mice (P ≤ 0.0236). Collectively, the expression of Sig-1r in the 3×TG-AD mice brain significantly decreased with the progression of AD. We further determined the expression of Sig-1r in the brain of 3×TG-AD mice aged 8-10 months, and the results showed that Sig-1r in the 3×TG-AD mice was lower than that in the WT mice (Fig.4.), which was consistent with previous studies demonstrating a large decrease and loss of Sig-1r in the brain regions of the early stage AD patients [2]. Therefore, 8-10 -month-old 3xTg-AD mice were selected in this study.

**
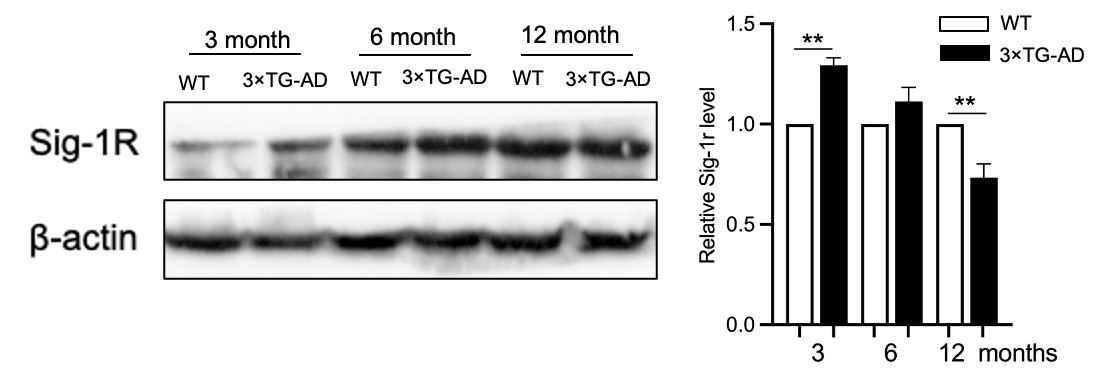
**

**Fig. S3** Sig-1r in the hippocampus of 3×TG-AD mice significantly decreased with the progression of AD. The expression of the Sig-1r in the mice brain was determined by using western blot. The qualification of expression was relative to match-aged WT mice. Data was expressed as mean ± SEM (n = 3 mice per group) and analyzed using unpaired T-test. **P ＜ 0.01.

**2.4 The effect of DMT on the cognitive dysfunction in 3×TG-AD mice.**

There was no significant difference in the improvement of the two parameters, time in platform and entries in platform, by DMT treatment between female and male mice (Fig. S4).


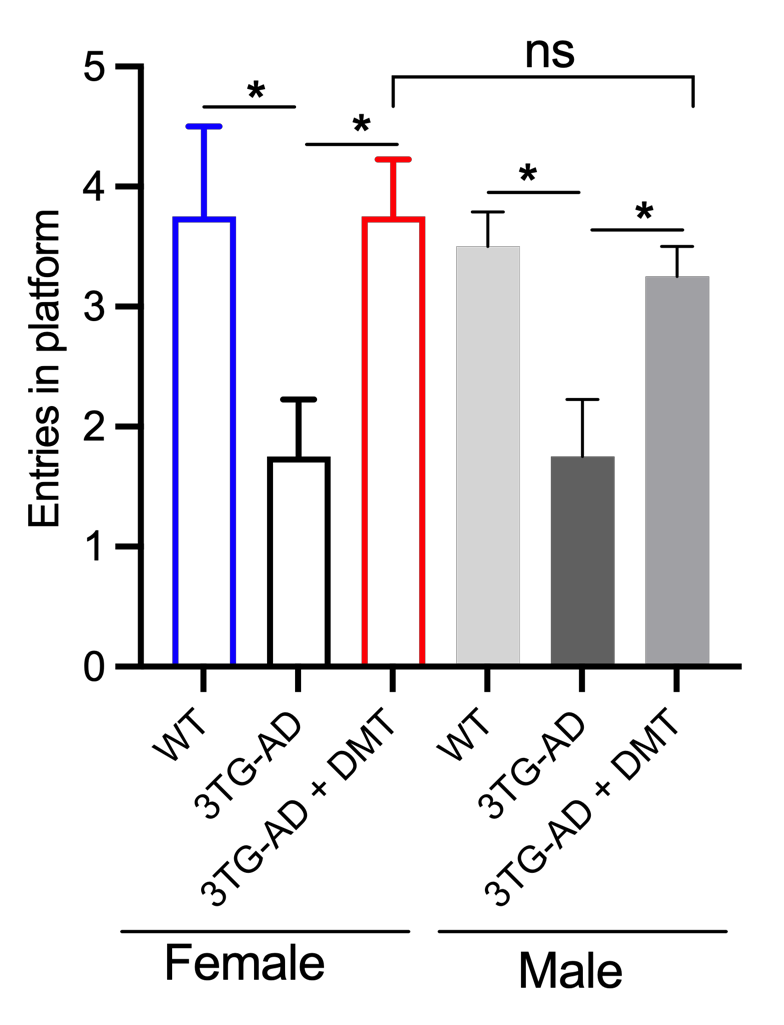


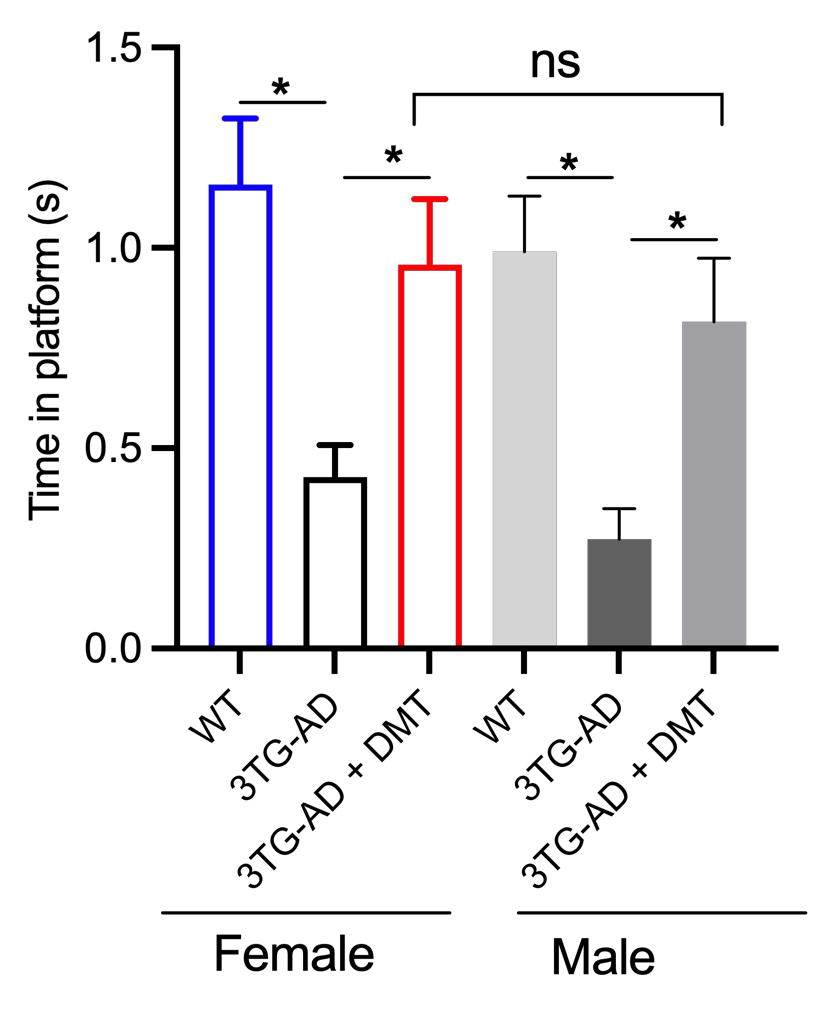


**Fig. S4** There was no significant difference in the improvement of cognitive dysfunction by DMT treatment between female and male mice.

**3. Details of the antibodies in the western blot**

**Table S1.** Antibody information for western blot.

| Antibody | Host | Manufacture | Dilution |
| --- | --- | --- | --- |
| PACS2 | Rabbit | Proteintech | 1:1000 |
| PSS1 | Rabbit | Proteintech | 1:1000 |
| MFN2 | Rabbit | Proteintech | 1:1000 |
| Sig-1r | Mouse | Sant Cruz | 1:1000 |
| VAPB | Rabbit | Abclonal | 1:1000 |
| CHOP | Rabbit | Abclonal | 1:1000 |
| β-actin | Mouse | Proteintech | 1:2000 |

**Reference**

1. Oddo, S., et al., *Amyloid deposition precedes tangle formation in a triple transgenic model of Alzheimer’s disease.* Neurobiology of aging, 2003. **24**(8): p. 1063-1070.

2. Hedskog, L., et al., *Modulation of the endoplasmic reticulum–mitochondria interface in Alzheimer’s disease and related models.* Proceedings of the National Academy of Sciences, 2013. **110**(19): p. 7916-7921.

**Gels and Blots image(s)**

1. PACS-2


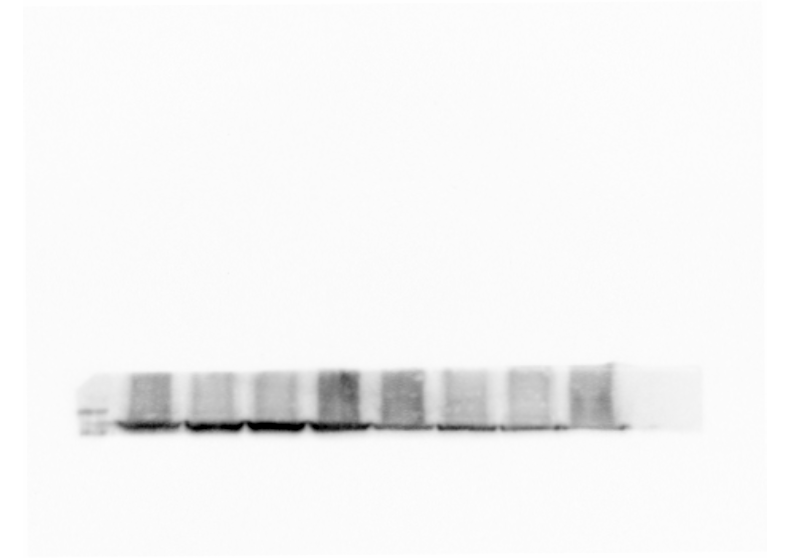


TG+DMT

TG

DMT

WT


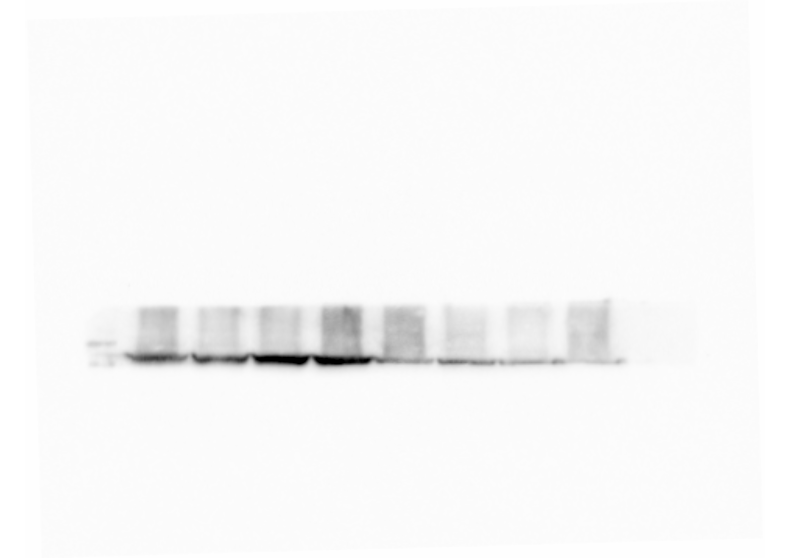


TG+DMT

TG

DMT

WT


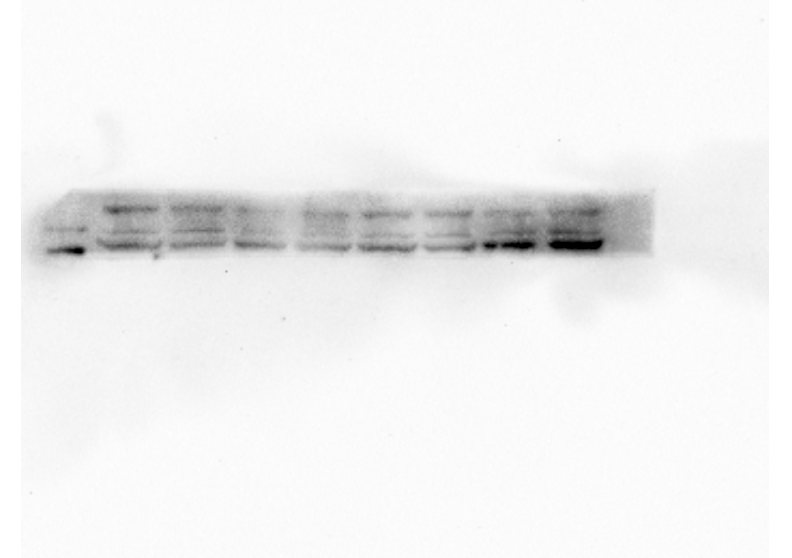


TG+DMT

TG

DMT

WT

2. MFN-2


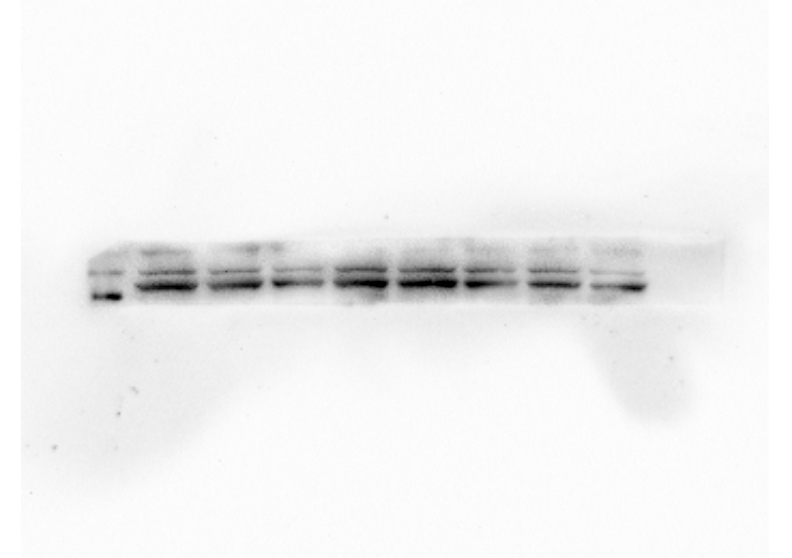


TG+DMT

TG

DMT

WT

TG+DMT

TG

DMT

WT


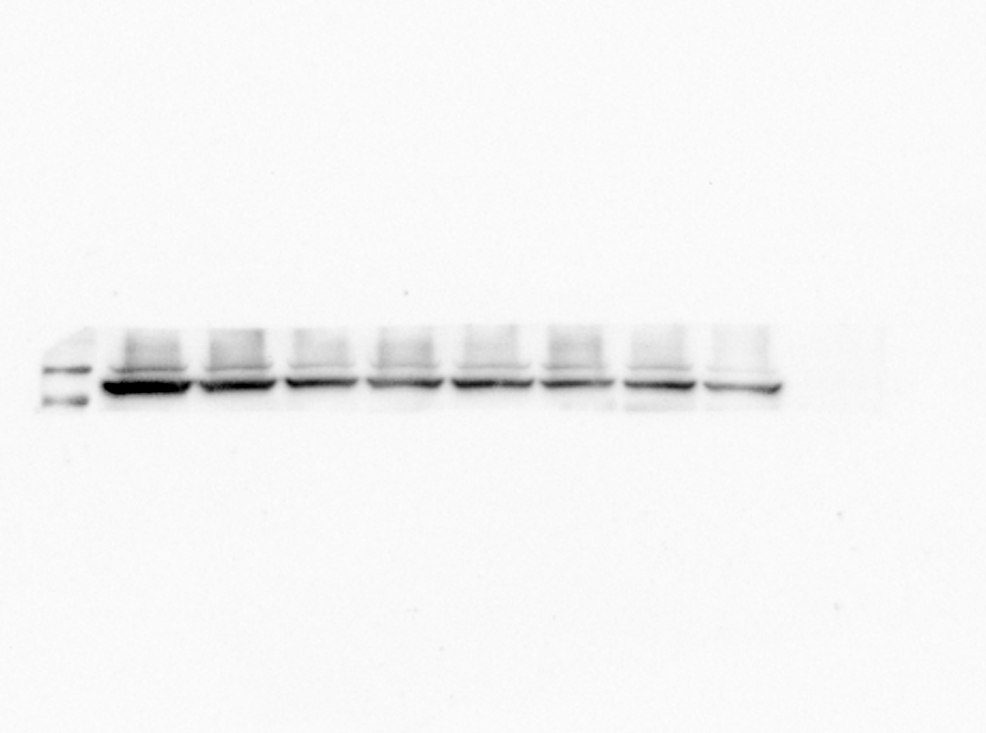


TG+DMT

TG

DMT

WT

3. PSS1


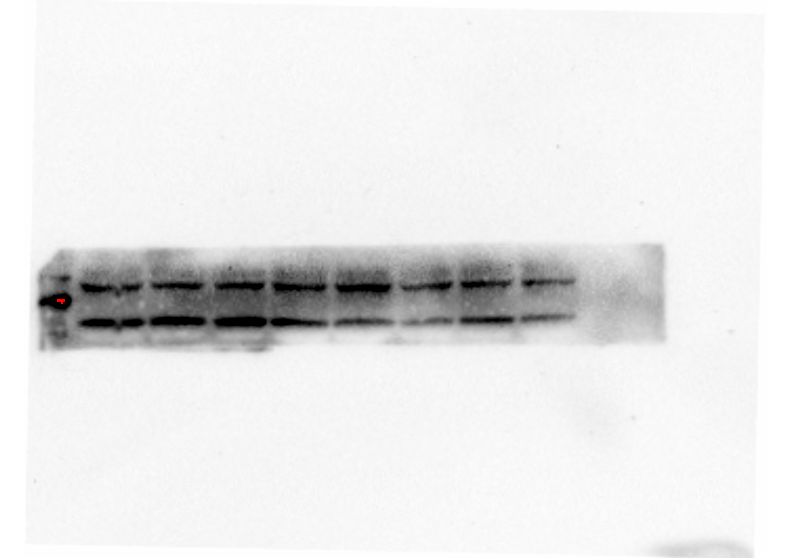


TG+DMT

TG

DMT

WT

WT

DMT

TG

TG+DMT


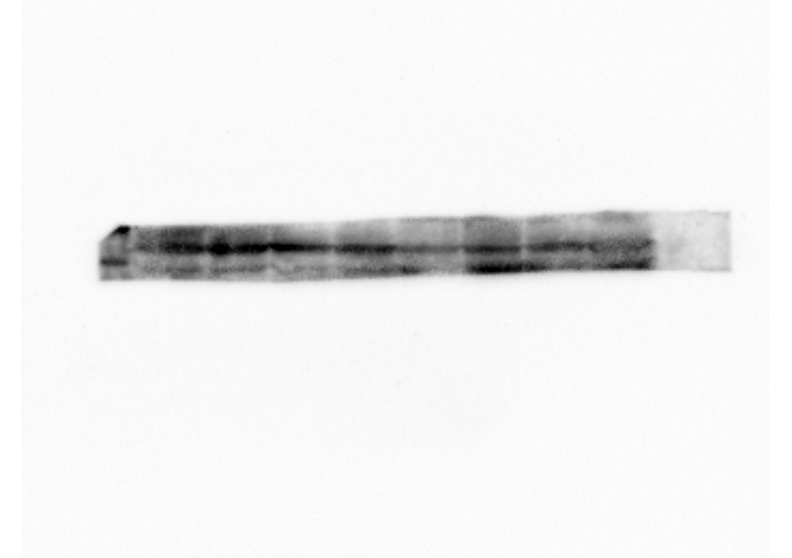


4. VAPB


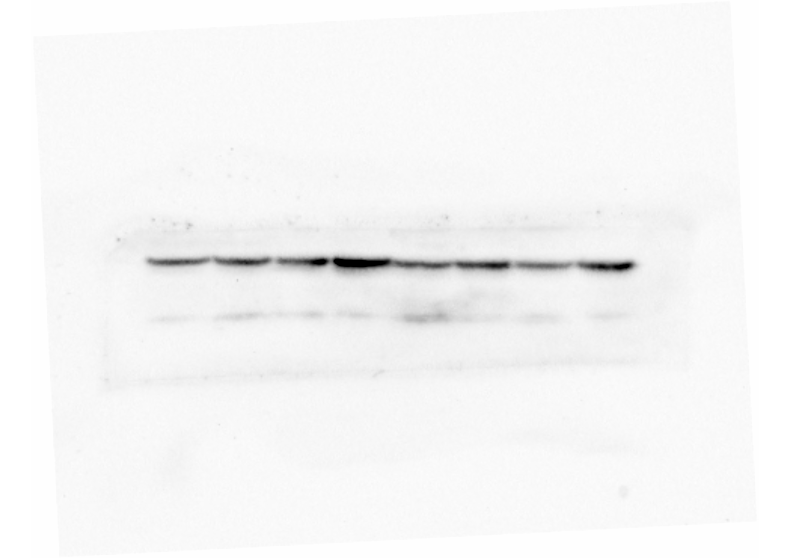


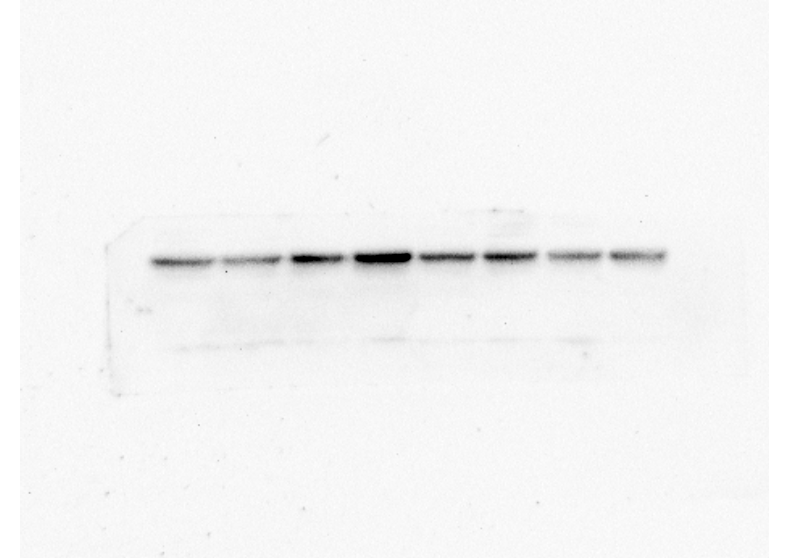


5. Sig-1R


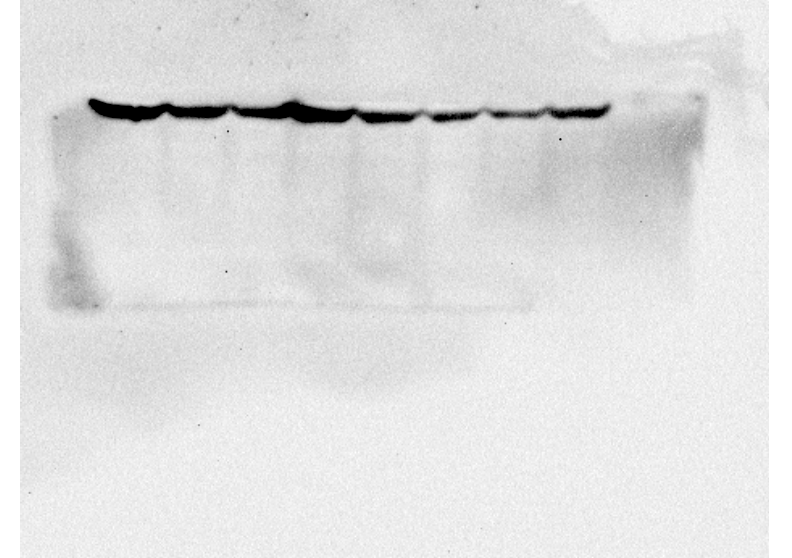


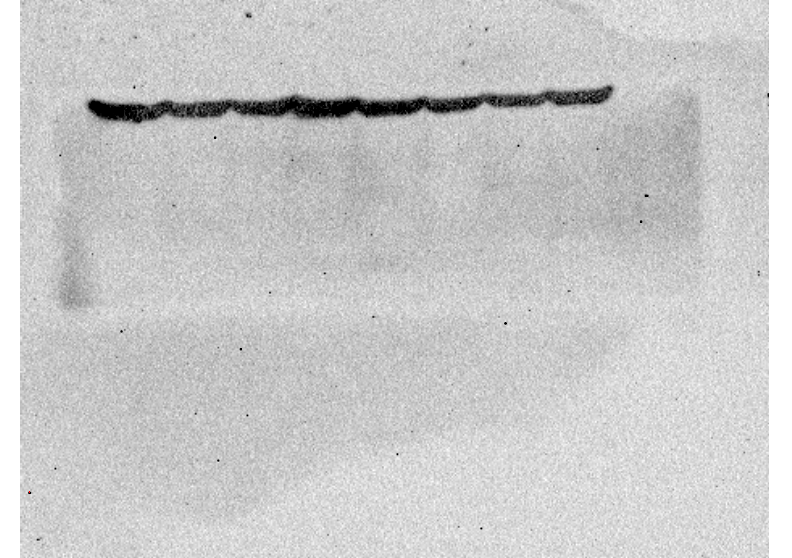


6. CHOP


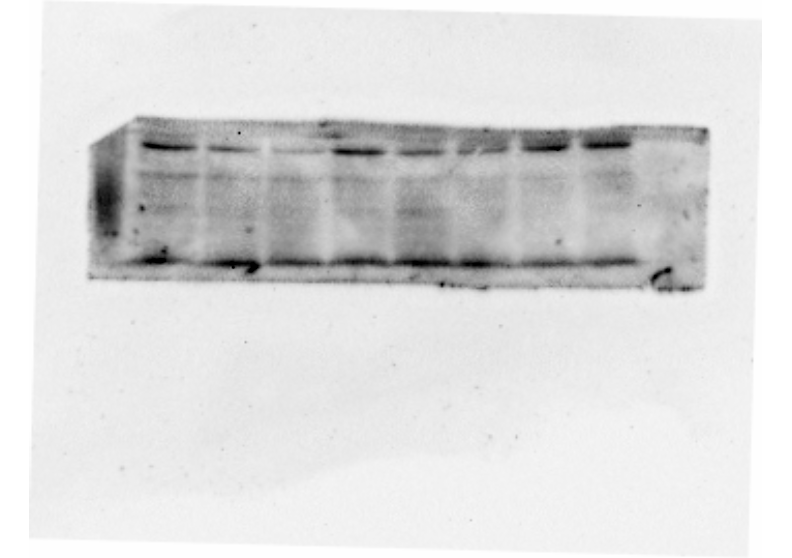


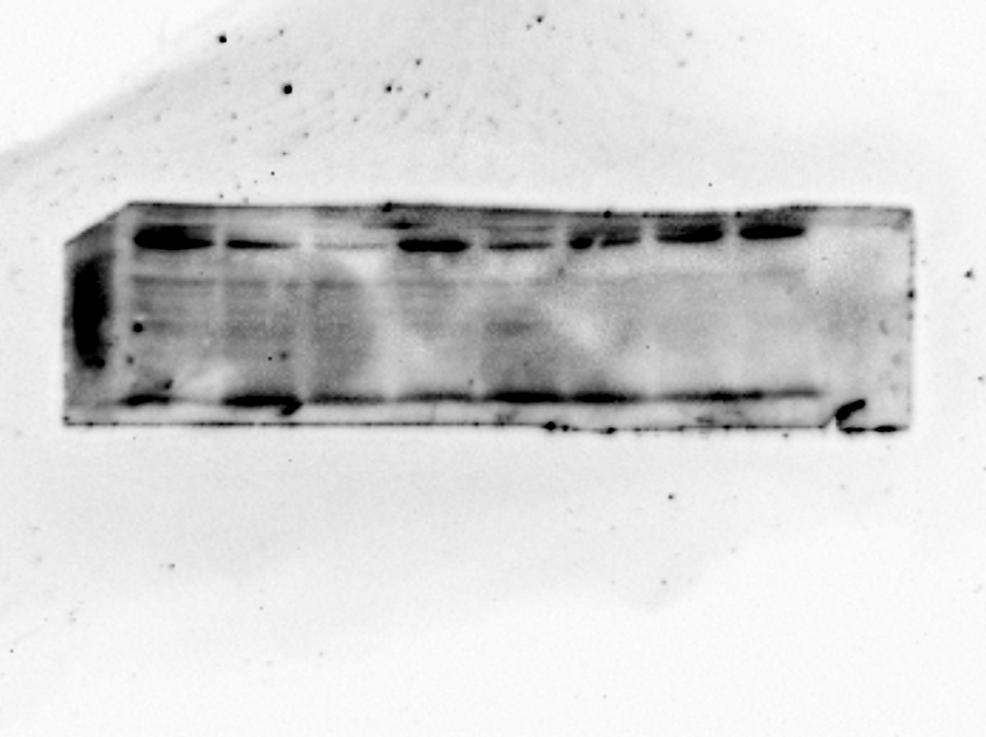


7. β-actin


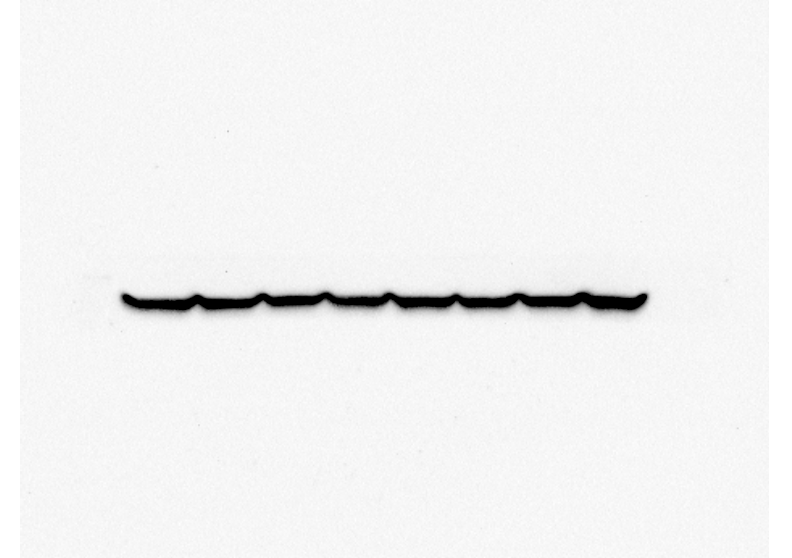


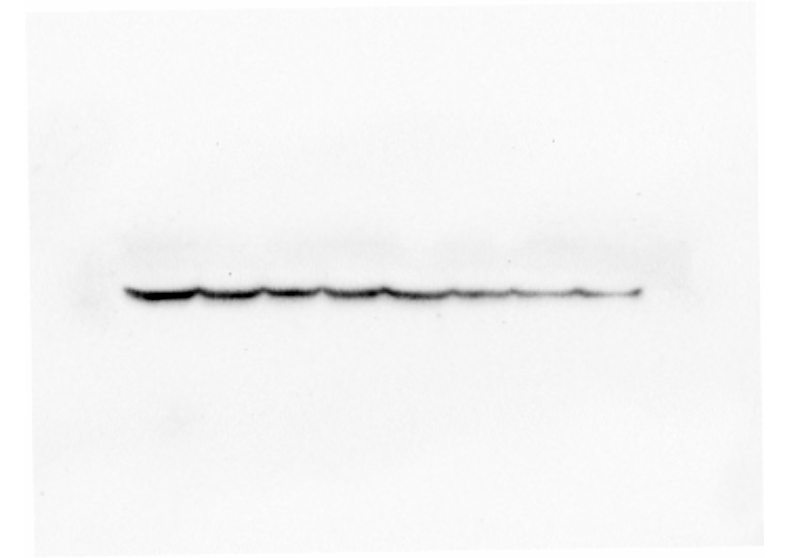

Supplement: Supplementary file 1 — Supplementary Material 1. [file 13195_2024_1462_MOESM1_ESM.docx]
